# Genome-wide variations in a natural isolate of the nematode *Caenorhabditis elegans*

**DOI:** 10.1186/1471-2164-15-255

**Published:** 2014-04-02

**Authors:** Ismael A Vergara, Maja Tarailo-Graovac, Christian Frech, Jun Wang, Zhaozhao Qin, Ting Zhang, Rong She, Jeffrey SC Chu, Ke Wang, Nansheng Chen

**Affiliations:** 1 Department of Molecular Biology and Biochemistry, Simon Fraser University, 8888 University Drive, Burnaby, British Columbia V5A 1S6, Canada; 2 School of Computing Science, Simon Fraser University, 8888 University Drive, Burnaby, British Columbia V5A 1S6, Canada

**Keywords:** *C. elegans*, Natural isolate strain, Next-generation DNA sequencing, Genomic variation, Loss-of-function, Large insertion, Compound variation

## Abstract

**Background:**

Increasing genetic and phenotypic differences found among natural isolates of *C. elegans* have encouraged researchers to explore the natural variation of this nematode species.

**Results:**

Here we report on the identification of genomic differences between the reference strain N2 and the Hawaiian strain CB4856, one of the most genetically distant strains from N2. To identify both small- and large-scale genomic variations (GVs), we have sequenced the CB4856 genome using both Roche 454 (~400 bps single reads) and Illumina GA DNA sequencing methods (101 bps paired-end reads). Compared to previously described variants (available in WormBase), our effort uncovered twice as many single nucleotide variants (SNVs) and increased the number of small InDels almost 20-fold. Moreover, we identified and validated large insertions, most of which range from 150 bps to 1.2 kb in length in the CB4856 strain. Identified GVs had a widespread impact on protein-coding sequences, including 585 single-copy genes that have associated severe phenotypes of reduced viability in RNAi and genetics studies. Sixty of these genes are homologs of human genes associated with diseases. Furthermore, our work confirms previously identified GVs associated with differences in behavioural and biological traits between the N2 and CB4856 strains.

**Conclusions:**

The identified GVs provide a rich resource for future studies that aim to explain the genetic basis for other trait differences between the N2 and CB4856 strains.

## Background

*C. elegans* is a model organism that has been widely used for biomedical research, shedding light on diseases such as Alzheimer [1] and cancer [2]. The genome of this hermaphrodite nematode species was the first one published of a multicellular animal [3] and its assembly and annotation is arguably one of the best of the multicellular organisms available today. The *C. elegans* reference genome sequence corresponds to the N2 strain, which was obtained from mushroom compost in Bristol, England, and later provided by Ellsworth Dougherty to Sydney Brenner in 1964 [4]. *C. elegans* populations can be found worldwide in North Africa, Europe, North America, Australia and islands such as Hawaii and Madeira [5]. Even though most *C. elegans* genetic studies have used the N2 background, the genetic and phenotypic differences of *C. elegans* among populations as found in different habitats has encouraged researchers to explore the natural variation of the nematode [6], which can be directly applicable to the understanding of human variation [7]. Genetic studies among different local [8-10] and global populations [11] have demonstrated that there is a low genetic diversity of this selfing species. This genetic diversity is 20× lower than that of *D. melanogaster*[6] or other obligately outcrossing members of the same genus [12], and comparable to that of human populations [6]. In general, the genetic diversity found within local populations is very close to that found among individuals located in different continents, with a likely explanation being the anthropogenic nature of *C. elegans* together with a metapopulation dynamics of bottlenecks and recolonisation of the habitat [13,14].

A strain that has been found to be one of the most genetically distant to the N2 strain is CB4856 [15,16], which was isolated in 1972 from a pineapple field in Hawaii [17]. In contrast to other isolates, this strain presents a large number of polymorphisms that are not found in any other populations [15] and it has been used in surveying mutations and studying natural selection in evolution [18], albeit allelic differences are likely to exist due to domestication during laboratory maintenance [19]. The considerably large number of polymorphisms found genome-wide (SNPs, and small InDels) made of this strain a good resource for gene mapping [20-22]. Lately, a new method based on confirmed SNPs between the CB4856 and N2 strains, called SNP-CGH mapping, has been proposed for the mapping of phenotypic traits [23]. Also, N2 and CB4856 backgrounds have been used for the generation of genetic tools such as Recombinant Inbred Lines (or RILs) [24,25], Recombinant Inbred Advanced Intercrossed Lines (RIAILs) [26] and Nearly Isogenic Lines (or NILs, also known as introgression lines) [27].

In addition to the usefulness of the polymorphic nature found between CB4856 and N2, these two strains present a number of differences in biological and behavioural traits such as copulatory plug formation [17,28], intake of O_2_ and CO_2_[29-31], temperature-size rule [32], germline RNAi [33], response to benzaldehyde [34], thermal migration [35], pathogen susceptibility [36], biofilm resistance in the presence of *Yersinia*[37], and social behaviour and food response [38,39]. Understanding the molecular basis of these and other biological differences is invaluable for annotating genes in *C. elegans*, which is a popular model organism for biomedical studies. For example, a missense mutation in gene *npr-1* is associated with differences in the response to CO_2_ and O_2_[29-31], social behaviour and food response [38,39] and susceptibility to pathogens [36]. Other examples are an early stop codon in *ppw-1* gene, which is associated with differences in germline RNAi [33], a missense mutation in *tra-3* gene, associated with differences in the temperature-size rule proper of ectotherms [32], the disruption of gene *plg-1* by an LTR-retrotransposon in the N2 background, associated with differences in copulatory plug formation [28] and the deletion of an exon in gene *glb-5*, associated together with *npr-1* with differences in the intake of O_2_ and CO_2_[29,30]. Although many lesions responsible for the phenotypic differences have been found, other known traits that present differences, such as the egg-laying behaviour or response to odorants [34] don’t have an identified genetic basis. Additionally, genes that don’t present genetic differences may also be associated to differences in traits by, for example, changes in gene dosage in one strain over the other due to epigenetic alterations.

Whole genome sequencing (WGS) and resequencing of *C. elegans* strains using second-generation technologies have gained increasing popularity as a fast and cost-effective method for understanding the genetic differences among wild isolates [40], laboratory strains [41], mutant strains [42-45], and mutation-accumulation (MA) lines for the study of mutational processes that lead to deleterious mutations [46] as well as fitness recovery through beneficial compensatory mutations [47]. In particular, no study has focused so far on the genome-wide genetic differences between the CB4856 and N2 strains based on WGS using second-generation sequencing technologies. Still, previous studies based on oligonucleotide array comparative genomic hybridization (oaCGH) have reported large copy number differences between these two strains [48,49], estimating that ~2% of the genes in the Hawaiian strain are deleted compared to the N2 strain. The oaCGH approach has a number of drawbacks, such as a limited resolution for the length of the InDels, no base pair level breakpoint resolution for the InDels detected, bias towards exonic regions of unique DNA content, and false positives in regions with a high content of SNPs and small InDels, where hybridization of the probe is not possible. Overcoming these drawbacks is essential for a clear and thorough understanding of the genomic differences between the Hawaiian and the N2 strain, since most of the genetic basis of phenotypic variants have been found to be small variations (as described above) and there are previous reports of highly polymorphic regions impacting both exonic as well as non-exonic segments of the *C. elegans* genome [50].

In this study, we have sequenced the CB4856 genomic DNA using Roche/454 and Illumina GA platforms. We show that the combined approach in which the strengths of both sequencing methods are used for the detection of GVs provides an accurate way of detecting single nucleotide variants (SNVs) and small insertions and deletions (small InDels) in highly variable and homopolymeric regions, as well as a basepair-level resolution of the detection of large deletions, insertions and compound variations. We also assessed the impact of all GVs on protein-coding genes by carefully considering all co-occurring GVs on a given transcript as well as the nature of the genes involved. We have not attempted to identify copy number variations between N2 and CB4856 in this study.

## Results

To identify genomic variations (GVs) between the genome of the N2 strain of *C. elegans* (version WS210 hosted at WormBase [51], used as reference) and the Hawaiian strain (CB4856), we have sequenced the CB4856 genome using the Roche 454 genome sequencer FLX system [52] and Illumina GA. The rationale behind this is that both sequencing technologies provide complementary strength: on the one hand, 454 reads provide the length necessary to detect large GVs such as insertions and deletions that cannot be found within the alignment of a read, but between two aligned segments (i.e., **h**igh-scoring **s**egment **p**air, HSPs) of a same read; on the other hand, Illumina reads provide the necessary coverage for reliably detecting SNVs and small InDels. Also, as shown below, the length of 454 reads proves useful for detecting SNVs and small InDels in highly variable regions, for which the alignment with Illumina reads is not feasible.

### Roche 454 genomic DNA sequencing

Our computational procedure for the identification of GVs based on 454 reads consists of two steps. First, the 1,237,732 reads obtained with 454 were aligned to the *C. elegans* reference genome (version WS210) using the Smith-Waterman-based program cross_match (http://www.phrap.org). The reads have an average length of 340 base pairs (bps) (median of 372 bps), and the alignment on the *C. elegans* reference genome achieves a 4× median depth. Most reads (637,016 or 51.5%) are aligned with a single HSP, which may contain SNVs as well as small insertion/deletions (InDels). Still, a large number generated two or more HSPs (585,805, or 47.2%), which suggests that larger GVs are occurring in addition to the SNVs and small InDels within each HSP.

To take full advantage of the long 454 reads for identifying GVs, we developed and applied our variation discovery program called variationBlast (described in Materials and Methods). Using variationBlast we categorized 1,146,783 reads as unique and 76,038 reads as non-unique (see Methods). The remainder 14,911 reads did not generate any alignmnents, which could be explained by sequences specific to the Hawaiian genome or to hypervariable regions. Based on the unique reads reported by variationBlast, we detected SNVs, small InDels, large insertions, deletions and compound variations (see below).

### Illumina Solexa genomic DNA sequencing

The detection of GVs based on Illumina reads for the Hawaiian strain was done as follows. First, the 85,494,844 Illumina reads (of 101 bps in length) were aligned in a paired-end manner (42,747,422 pairs in total) against the WS210 release of the *C. elegans* genome using SSAHA2 [53]. Of these, 76,629,083 reads (or 89.6% of the total) were mapped to the genome, generating a median depth of 67×. Second, based on this alignment, VarScan [54] was used to detect SNVs and small InDels. Those reads that mapped only partially to the genome (this is, either a 5′ or a 3′ flanking region of its sequence doesn’t align) were used as input to detect large deletions in the same manner done with 454 reads (see Methods for details).

### Identification and assessment of SNVs

**Figure 1:**
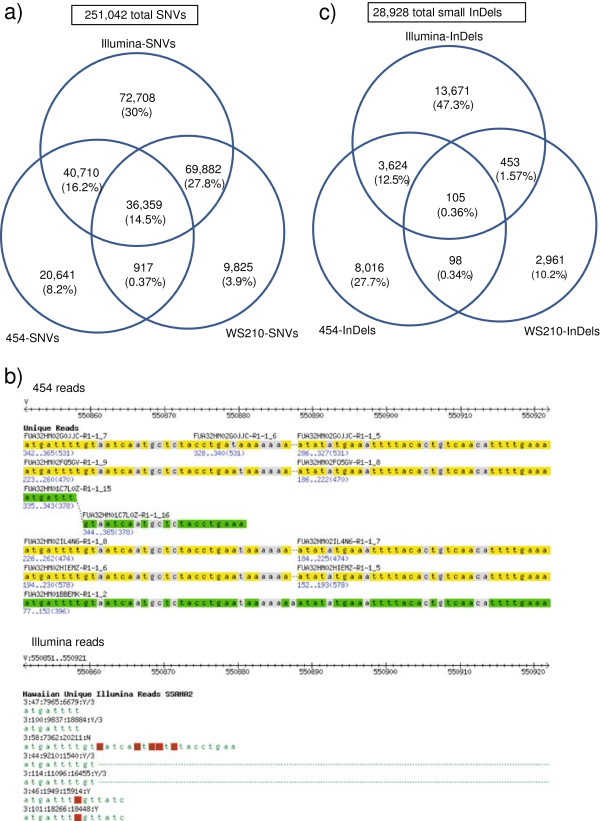
**Agreement of SNVs and InDels found with different methodologies. a)** Venn diagram of SNVs found with different methodologies. **b)** Example of a highly variable region, alignment of 454 reads (top) and Illumina reads (bottom). The region corresponds to V:550851..550921 in the left arm of chromosome V. Reads in green colour indicate those aligned on the positive strand, whereas reads in yellow indicate those aligned on the negative strand. Base pair differences between Hawaiian reads and the reference genome are depicted in grey for the 454 reads and in red for the Illumina reads. **c)** Venn diagram of small InDels found with different methodologies.

The strategy based on 454 reads and variationBlast yielded 98,664 SNVs (hereafter called 454-SNVs) whereas that based on Illumina reads and VarScan yielded 219,712 SNVs (Illumina-SNVs). Additionally, WormBase WS210 lists 116,999 SNVs (WS210-SNVs). Merging the three datasets generates a total of 251,042 SNVs (Additional file 1), after excluding 53 SNVs due to inconsistencies in the nucleotide variant between two or among all datasets (Additional file 2). As expected due to its deep coverage, Illumina reads contribute for the vast majority (87.5%) of the total SNVs (Figure 1a).

Inspection of the 454 aligned reads on those coordinates that are specific to Illumina-SNVs shows that they are missed by 454 due to low coverage (either no reads or a single read aligned) and due to conflicting reads at the same sites. Further analysis of SNVs specific to 454-SNVs shows that many fall into highly variable regions that don’t allow for an alignment with Illumina reads and SSAHA2 (Figure 1b). This is a valuable contribution of the length of the 454 reads to the detection of SNVs, since otherwise these regions would be seen as gaps in the Hawaiian genome compared to the reference.

**Figure 2:**
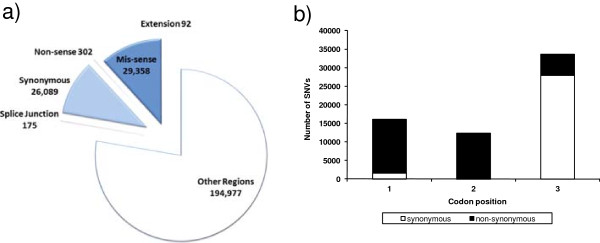
**Categorization of SNVs by (a) feature impacted, and (b) codon bias.** For the categorization on protein-coding genes in **(a)**, 71 SNVs were left out since their category varies on different transcripts.

The other explanation found for those SNVs that are unique to 454 is that they are supported by Illumina reads, but are discarded either by the minimum read coverage or by the variant frequency threshold. This latter reason also applies to SNVs specific to WS210. Of the 251,042 total SNVs, transitional substitutions are slightly more frequent than transversional substitutions (53.5% versus 46.5%), which is expected and consistent with previous observations in *Caenorhabditis*[20,21] as well as other species [55,56]. Although the majority of the detected SNVs fall in non-coding regions (we refer to a non-coding region as any region that is not a protein-coding exon or a splice junction), a large number of them (56,016, or 22.3%) fall within protein-coding exons or splice junction sites, suggesting that SNVs have a huge potential to impact the structure and function of protein-coding genes (Figure 2). We also assessed the impact of SNVs on individual spliced forms because same SNVs can have differential impact on different spliced forms of a same gene. Altogether, 49 SNVs belong to two or more categories by impacting different spliced forms differently. For example, the SNV in coordinate V:17774670 (T > A) generates a missense substitution for spliced form C47A10.5a but a non-sense mutation for spliced form C47A10.5b. For 41 of these SNVs, the difference between spliced forms of a same gene corresponds to a synonymous SNV in one spliced form that is also missense in another spliced form.

**Figure 3:**
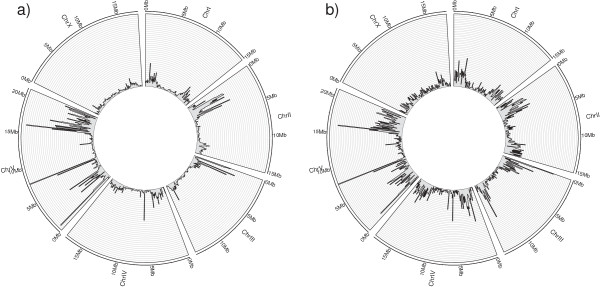
**CIRCOS representation of a) SNVs genomic distribution, b) small InDels genomic distribution.** The figures were generated using the histogram option, with a bin size of 100,000 bps.

Interestingly, more than half of the SNVs in protein-coding exons are non-synonymous (including both non-sense and missense SNVs), suggesting that some regions of the genome are undergoing strong positive selection (Figure 2a). For those SNVs that fall within protein-coding exons, there is a bias for SNVs in the third position compared to the first and second position (Figure 2b). Also, the occurrence of SNVs is higher in the arms of the autosomal chromosomes compared to the center, with a rather uniform pattern for the X chromosome, as shown in the genomic distribution of SNVs illustrated using the software Circos [57] (Figure 3a). These observations are in agreement with previous studies on the genomic architecture of *C .elegans* N2 based on strain comparisons [15,40] as well as inter-species comparisons [58-61].

**Figure 4:**
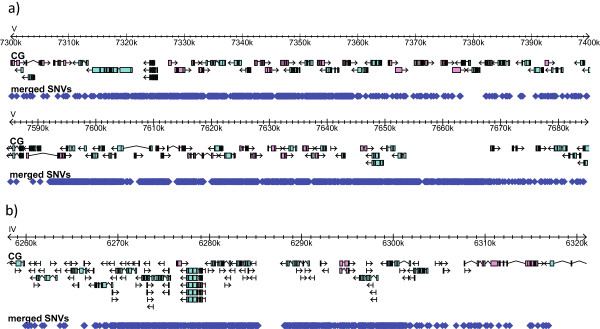
**Impact of SNVs on chemosensory genes. a)** Two 100 kbp regions in the center of chromosome V. **b)** 50 kbp region in the center of chromosome IV.

From Figure 3a we can observe that the left arm of chromosome II and chromosome III as well as both arms of chromosome V have the highest density of SNVs. Unexpectedly, and in addition to these large regions in the arms of chromosomes, two smaller regions in the center of chromosome V spanning ~100 kbp each (chr V, 7300 kbp to 7400 kbp and 7590 kbp to 7690 kbp) and one region in the center of chromosome IV spanning ~60 kbp (chrIV, 6260 kbp to 6320 kbp) also have a very high density of SNVs. Inspection of these regions shows that these are mostly chemosensory genes (Figure 4), which have been demonstrated to be actively evolving [62]. In fact, chemosensory genes are among the most rapidly evolving genes in *Caenorhabditis* species, as demonstrated by comparative analysis of chemosensory gene families [60,63-65].

One-third of all *C. elegans* genes (7,243, or 35.7%) contain one or more missense SNVs, whereas 257 genes (1.3%) carry one or more non-sense SNV. Non-sense SNVs accumulate preferentially in the 3′ end of the coding sequence (Additional file 3), suggesting that many genes containing non-sense SNVs might still be functional. In contrast, missense SNVs distribute rather uniformly along the coding sequence (Additional file 3). The large number of non-synonymous SNVs suggests a significant impact on protein-coding genes for this type of GV.

In order to evaluate the potential functional impact that missense SNVs have on protein-coding genes, we examined, for each corresponding amino acid substitution, the associated Grantham Score (GS) [66]. This score predicts the difference between two amino acids in terms of composition, polarity and molecular volume. Based on the categorization provided by [67], the differences can be regarded as: Radical (GS > 150), Moderately Radical (GS between 101 and 150), Moderately Conservative (GS between 51 and 100) and Conservative (GS between 1 and 50). Based on this, we find that 5.5% of the amino acid substitutions can be regarded as Radical (1,439 sites), 13.6% as Moderately Radical (3,557 sites), 42.8% as Moderately Conservative (11,183 sites) and 38.1% as Conservative (9,942 sites). Taking together the percentage of Radical and Moderately Radical substitutions, approximately 1 in 5 substitutions are predicted to generate an important change on protein structure, and hence likely function.

### Identification and assessment of small InDels

In this project, small InDels are defined as insertions and deletions that cause gaps in local sequence alignments obtained using cross_match, for 454 reads (http://www.phrap.org), or SSAHA2 [53] when aligning Illumina reads. Using 454 reads and variationBlast, we found 11,858 small InDels (hereafter called 454-InDels) whereas with Illumina reads and VarScan we found 17,863 small InDels (Illumina-InDels). Additionally, WormBase WS210 provides 3,629 small InDels (WS210-InDels).

Merging of the three datasets generates a total of 28,928 small InDels (Figure 1c and Additional file 4), after excluding 17 small InDels due to inconsistencies in sequence among datasets (Additional file 5). As expected due to its deep coverage, and as it was observed for SNVs, Illumina reads contribute for the majority (61.7%) of the total small InDels. The overlap between Illumina and 454 is much lower for InDels than for SNVs (12.9% *vs.* 30.7%, Figure 1). Inspection of InDels unique to one platform reveals two main reasons for this discrepancy. First, the majority of the Illumina-InDels are found adjacent to homopolymeric regions (Additional file 6). Since sequencing of homopolymers is a known issue for 454 reads [52], small 454-InDels within such regions were filtered out by our methodology for homopolymers of length 5 bps or larger. The high presence of small InDels in homopolymeric regions have also been reported previously for the Pasadena strain (*i.e.*, CB4858) when comparing it to the N2 strain [40]. This finding further illustrates the importance of sequencing the Hawaiian genome with both 454 and Illumina methodologies; in addition to the sensitivity gained with 454 reads for highly variable regions, the accuracy of Illumina reads at homopolymeric regions greatly improves the detection and estimation of the number of small InDels, which would have been greatly underestimated otherwise. The second source of disagreement between these datasets is that different strategies for alignment of reads have an impact on the upper threshold for what is regarded as a small InDel. For 454-InDels, their length distribution goes up to 39 bps, whereas for Illumina-InDels their length distribution goes up to 13 bps only (Additional file 7). Additional reasons for uniqueness of Illumina-InDels, 454-InDels and WS210-InDels are in close agreement with those found for SNVs.

The total small InDels range in length from 1–39 bps, have a median of 1 bp, and the majority fall outside of exonic regions (Additional file 8a). For those InDels of length 2 bps or larger, there is a higher frequency of those that don’t generate frameshift compared to those that do, which is not observed for small InDels that fall in non-exonic regions (Additional file 8b). This suggests that small InDels that do not cause frameshifts on protein-coding genes are more tolerated through evolution than those that do.

Still, 1,139 genes (or 5.6% of the total genes, with associated 1,284 spliced forms) are impacted by small InDels, with 702 genes (795 spliced forms) having their ORF disrupted, in many cases at the 3′ end of their coding sequence (Additional file 9). This suggests, in the same way as for SNVs, that many genes containing disruptive small InDels might still be able to keep their functionality.

The frequency of InDels is higher in the arms of the autosomal chromosomes compared to the centres (Figure 3b). In contrast, the distribution of small InDels is rather uniform on the X chromosome. In general, there is a striking agreement between the distribution of SNVs and InDels, including those regions with a high frequency of mutations in the center of chromosome IV and chromosome V that contain mostly chemosensory genes.

Next, we describe the identification of large deletions, insertions, and compound variations. Since different patterns of aligned reads were observed, we have defined different types of insertions and compound variations (Table 1).

**Table 1:** Large structural variations defined in this study

**Type of variation**	**Definition**
Large deletion	A genomic sequence revealed as a gap between adjacent and co-linear aligned segments or HSPs (Figure 5a)
Type-A insertion	An unaligned portion of a read that is flanked by two HSPs of the same read, (Figure 6a)
Type-B insertion	The flanking regions of two or more convergent reads are not aligned to the genome. These unaligned flanking regions might represent the 5′ and 3′ ends of a putative large insertion (Figure 6c)
Deletions associated with type-A insertions	Co-occurring deletion and type-A insertion where the deletion is equal or larger than the type-A insertion at the same breakpoint (Figure 8a, left)
Type-A insertions associated with deletions	Co-occurring type-A insertion and deletion where the type-A insertion is larger than the deletion at the same breakpoint (Figure 8a, right)
Type-B insertions associated with deletions	Co-occurring type-B insertion and deletion where the type-B insertion pattern for which the convergent reads are at a distance larger than zero (Figure 8b)

### Identification and assessment of large deletions

**Figure 5:**
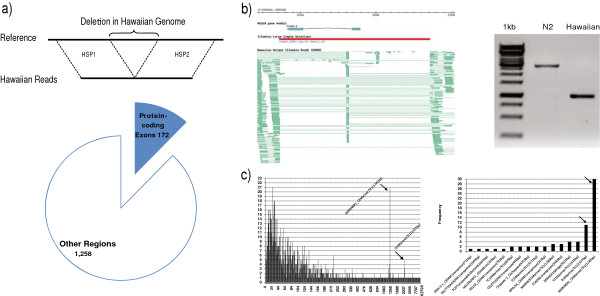
**Large deletion detection and distribution. a)** Illustration of large deletion detection and impact on protein-coding exons. **b)** Gbrowse visualization of a deleted gene, F42A6.5 and its experimental validation through PCR amplification. **c)** (Left) Distribution of large deletions reveals peaks associated with transposable elements. Two peaks around lengths 1,244 and 2,337 bps are explained by *Cemar1* and Tc3 transposable elements, respectively. **c)** (Right) Frequency of different types of transposable elements found deleted in the Hawaiian genome compared to reference. Name is provided as annotated in RepBase, with the length of the transposable elements shown in parenthesis.

We defined large deletions as genomic sequences revealed as gaps between adjacent and co-linear aligned segments (or HSPs). We identified large deletions using 454 reads and variationBlast on the HSPs generated with cross_match (Figure 5a). Compared to the reference genome, we found 533 deletions in the Hawaiian genome (hereafter called 454 large deletions). Applying the same idea on Illumina reads (see Methods) we found 1,334 deletions in the Hawaiian genome compared to the reference (hereafter called Illumina large deletions). Merging of the two datasets generates a total of 1,430 large deletions (Additional file 10), with 437 of the 533 large deletions obtained with 454 reads confirmed by Illumina large deletions (82% of the 454 large deletions). Hence, the procedure defined with cross_match and variationBlast on Illumina reads identified 93.3% of the total large deletions. As expected, large deletions found with 454 but not with Illumina are due to thresholds on the maximum depth allowed within the deletion as well as the minimum number of supporting reads (see Methods); large deletions found with Illumina but not with 454 are mostly due to low coverage with 454.

The 1,430 large deletions have a median length of 85 bps, and range from 4 bps to 62,795 bps in length, with 640 deletions (44.8%) equal or larger than 100 bps in length, and 151 deletions (10.6%) equal or larger than 1,000 bps. The majority of these deletions (88%) fall in regions without protein-coding exons, with 172 deletions impacting 206 protein-coding genes (Figure 5a). Of these 206 genes, 50 are entirely deleted (51 spliced forms deleted), 80 are truncated (84 spliced forms disrupted), 75 genes have preserved ORF (224 spliced forms), and 1 gene (F14D2.4) has one spliced form with its ORF preserved (F14D2.4b) whereas the other spliced form has its ORF disrupted (F14D2.4a). An example of a gene fully deleted in Hawaiian is F42A6.5, which has homology to human BRCA1, associated with breast cancer. This deletion, of 1,996 bps in length, is experimentally confirmed (Figure 5b).

Close inspection of the length distribution of large deletions reveals two peaks at lengths 1,244 and 2,337 bps (Figure 5c). Since such peaks can be indication of transposon activity, we performed a blastn search [68] of these deleted sequences against the RepBase 15.11 library for *C. elegans*[69]. All 1,244 bps deletions yielded matches with e-value < 1e-100 for MARINER2_CE, whereas all 2,337 bps deletions yielded matches with e-value < 1e-100 for Tc3, two Mariner/Tc1 elements. In order to assess the overall impact of transposon activity on the large deletions, we ran blastn of all deleted sequences against RepBase 15.11, searching for hits with evalue < 1e-100 and not allowing for differences between the length of the deletion and that of the transposable element to be larger than 10% of the length of the transposable element. In this way we found 70 large deletions ranging from 193 bps to 5,625 bps to be explained by transposable elements (Figure 5c, Additional file 11). 61 of these large deletions are larger than 1,000 bps, explaining 40.4% of deletions larger than 1,000 bps.

### Identification and assessment of large insertions

A striking advantage of using the Roche/454 sequencing method compared to other second-generation DNA sequencing methods that generate shorter reads is the potential to identify insertions in the target genome with breakpoints defined at the base pair resolution, as demonstrated in the Watson genome analysis [56]. Although paired-end reads generated using other second-generation DNA sequencing methods such as Illumina can be used to estimate the existence of insertions, the exact breakpoints are not defined. Since the detection of insertions is limited by read length, we define and identify large insertions of various sizes by examining the nature of unaligned segments between HSPs generated using cross_match.

**Figure 6:**
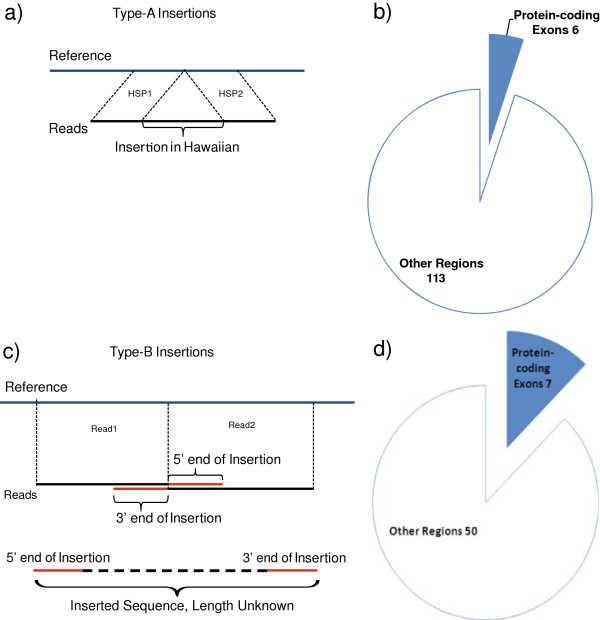
**Type-A and Type-B insertion detection and impact on protein-coding exons. a)** Type-A insertions are detected as unaligned regions of the reads found between two aligned regions. **b)** Impact of type-A insertions on protein-coding exons versus other regions. **c)** Type-B insertions are detected as convergent unaligned flanking regions of different reads. **d)** Impact of type-B insertions on protein-coding exons versus other regions.

If an unaligned portion of a read is flanked by two HSPs of the same read, then it is annotated as a type-A insertion (Figure 6a). These insertions are shorter than the read length, with their exact length, content and breakpoints readily defined. We identified 119 type-A insertions in the Hawaiian genome ranging from 12 bps to 288 bps in length, with a median length of 56 bps (Additional file 12). Of these insertions, 24 (20.2%) are equal or larger than 100 bps in length. The majority of these 119 type-A insertions (95%) fall in regions without protein-coding exons, with only six insertions impacting six protein-coding genes (Figure 6b). Evaluation of these six insertions on the impacted protein-coding genes shows that two of them preserve ORF (K05C4.3 and Y14H12A.1), whereas the other four disrupt the ORF (Y17G9B.8, C38C3.7, F21H7.14 and Y43F8C.18), preferentially at the 3′ end of the sequence, with exception of Y43F8C.18 whose disruption occurs in the first half of the coding sequence.

The limitations imposed by the read length for detecting large insertions drove us to define a separate strategy. If the flanking regions of two or more convergent reads are not aligned to the genome, then these unaligned flanking regions might represent the 5′ and 3′ ends of a putative large insertion (Figure 6c). We call this putative insertion a type-B insertion. As type-A insertions, the breakpoints of type-B insertions are clearly defined at the base pair resolution. However, in contrast to type-A insertions, type-B insertions are of unknown length and content without further assessment. Compared to the reference genome, we detected 57 type-B insertions in the Hawaiian genome (Additional file 13). The majority of these insertions (50) fall in regions without protein-coding exons, with seven insertions affecting seven protein-coding genes (Figure 6d).

**Figure 7:**
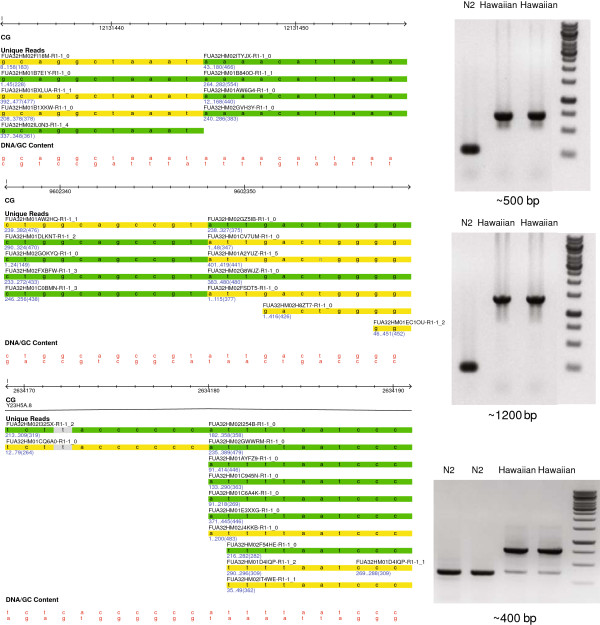
**Three type-B insertions validated experimentally.** For each case, the alignment of 454 reads is displayed at the left, and the gel of the resulting PCR reaction is shown at the right. The gels are loaded as follows: (lane 1) N2 control, (lanes 2 and 3) PCR product from CB4856, (lane 4) Fermentas 1 kb ladder. Green reads indicate those aligned on the positive strand, whereas yellow reads indicate those aligned on the negative strand.

There are two complementary approaches for defining the sequences of the type-B insertions. First, the unaligned reads can be assembled into contigs. Assembled contigs are then compared and aligned with the flanking regions of the insertion sites for the identification of insertions. Unfortunately, the assembly provided with the Roche/454 sequencing didn’t prove useful for this purpose, likely due to a short contig length (median of 1.3 kbp). Alternatively, we can examine the detected type-B insertions experimentally by PCR amplification of these insertions. We confirmed 3 candidate type-B insertions and identified the lengths of these insertions as ~400 bps, ~500 bps, and ~1.2 kbp (Figure 7). As expected, these type-B insertions are much larger than even the largest type-A insertion found, of 288 bps in length, and also to those large insertions found in the Watson genome [56], for which the largest insertion is 208 bps in length based on 250 bps 454 reads.

### Compound variations

**Figure 8:**
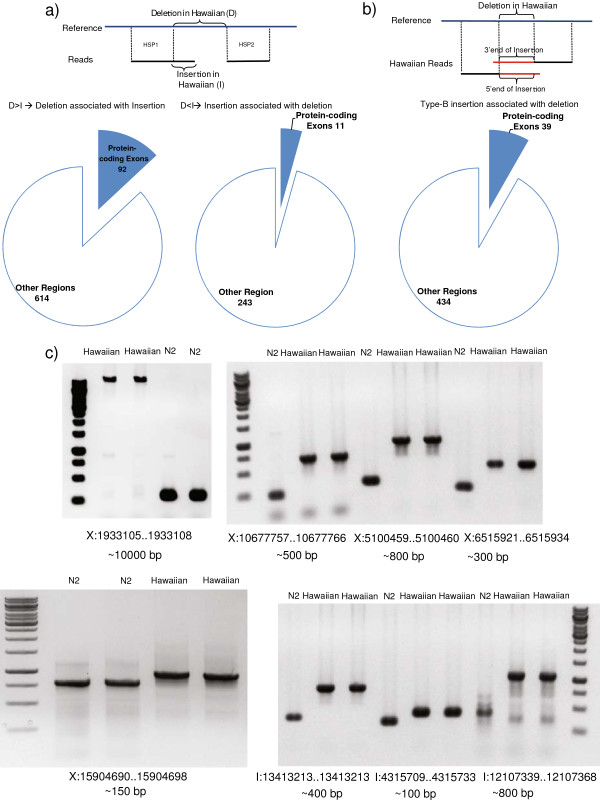
**Detection and validation of compound variations. a)** Illustration and distribution of compound deletions and type-A insertions. **b)** Illustration and distribution of type-B insertions with associated deletions **c)** Validated type-B insertions with associated deletions. The estimated length of the insertion is shown under each gel.

In addition to the events described above involving insertions and deletions, we have found a large number of variations with a co-occurrence of insertions (type-A or type-B) and deletions at the exact same breakpoints. We thus distinguish them from the previously described “simple” large insertion and deletion events and define three main categories of compound variations: (i) Deletions associated with type-A insertions, when the deletion is equal or larger than the type-A insertion at the same breakpoint (Figure 8a, left), (ii) type-A insertions associated with deletions, when the type-A insertion is larger than the deletion at the same breakpoint (Figure 8a, right) and (iii) type-B insertions associated with deletions, when there is a type-B insertion pattern for which the convergent reads are at a distance larger than zero (Figure 8b).

Compared to the reference genome, we found 706 deletions associated with type-A insertions in the Hawaiian genome (Figure 8a, left; Additional file 14). These deletions have a median length of 106 bps, and range from 11 bps to 56,263 bps in length, with 372 deletions (52.7%) equal or larger than 100 bps in length, and 46 deletions (6.5%) equal or larger than 1,000 bps. The associated insertions range in length from 1 bp to 311 bps, with a median length of 8 bps, and 48 insertions are equal or larger than 100 bps. The majority of these deletions (87%) fall in regions without protein-coding exons, with 92 deletions affecting 125 protein-coding genes (with corresponding 166 spliced forms). Of these 125 genes, 47 are entirely deleted, 40 are truncated with some coding region deleted in the Hawaiian genome, 37 preserve the ORF and one gene, C29F9.3, is such that its ‘a’ and ‘c’ spliced forms are fully deleted, whereas its ‘b’ spliced form has the ORF disrupted.

When the inserted sequence is larger than the deletion, we call it a type-A insertion associated with deletion. Compared to the reference genome, we found 254 type-A insertions associated with deletions in the Hawaiian genome (Figure 8a, right; Additional file 15). These insertions have a median length of 65 bps, and range from 13 bps to 358 bps in length, with 75 type-A insertions (29.5%) equal or larger than 100 bps in length. The associated deletions range in length from 1 bp to 339 bps, with a median length of 11 bps. The majority of these type-A insertions with their associated deletions (95.7%) fall in regions without protein-coding exons, with 11 of these variations impacting 11 protein-coding genes (with corresponding 11 spliced forms), 4 of them resulting in a disrupted ORF, and 7 of them having their ORF preserved. Manual inspection of the sequences inserted and deleted within these compound variations suggests that some of them correspond to small duplications at the breakpoints (data not shown).

In addition to the deletions associated with type-A insertions and vice versa, we also found 473 type-B insertions associated with deletions in the Hawaiian genome (Figure 8b; Additional file 16). As stated before, the type-B insertions detected in this work have no known content or length without further experimental assessment, but they are expected to be large insertions. The associated deletions range in length from 1 bp to 383 bps, with a median length of 17 bps. The majority of these type-B insertions and their associated deletions (91.8%) fall in regions without protein-coding exons, with 39 of them impacting 37 protein-coding genes (corresponding to 41 spliced forms). We selected and confirmed experimentally 8 candidates, providing inserted sequences ranging in length from 100–800 bps, with a particular case of a 10 kbp insertion (Figure 8c) in the Hawaiian genome. In addition to the cases validated for “simple” type-B insertions, these further prove the validity of this approach for detecting large insertions.

### Impact of GVs on protein-coding genes and loss-of-function mutations

**Figure 9:**
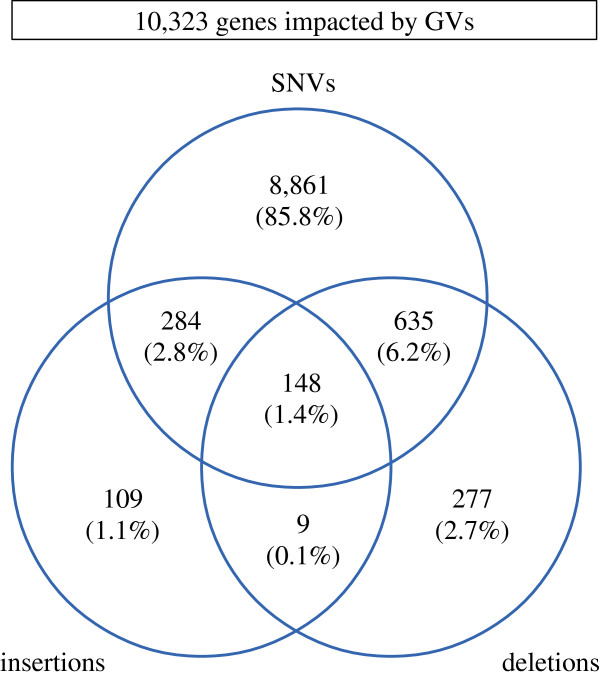
**Venn diagram of the impact of GVs on protein-coding genes.** Small insertions (resp. small deletions) and large insertions (resp. large deletions) are grouped under the same category for simplification of the diagram.

The detection of SNVs, insertions and deletions between the Hawaiian strain and the N2 reference strain makes evident the huge disruptive potential that these GVs have by themselves on protein-coding genes. Furthermore, hundreds of genes are simultaneously impacted by two or more of these GVs (Figure 9). Hence, if the impact of GVs on the functionality of protein-coding genes is to be analysed accurately, then all co-occurring GVs should be considered. We used our newly developed tool CooVar [70] to explore the disruptive potential of all GVs. In particular, we assessed the impact of co-occurring SNVs, small InDels, large deletions, type-A insertions, type-A insertions associated with deletions and deletions associated with type-A insertions on all protein-coding genes annotated for *C. elegans* release WS210. GVs involving type-B insertions were not included as they are of unknown content and length. After running CooVar, we found 10,323 genes, corresponding to 12,248 spliced forms, impacted by some kind of GVs (Additional file 17). Of these, (i) 93 genes are fully deleted (95 spliced forms), (ii) 1,128 genes have a disrupted ORF (1,244 spliced forms), (iii) 2,586 genes contain radical or moderately radical SNVs (2,889 spliced forms), (iv) 7,828 genes are impacted by GVs other than synonymous SNVs (9,094 spliced forms) and (v) 9,859 genes are impacted by synonymous SNVs (11,718 spliced forms). Within this last category, 1,340 genes were found to be under purifying selection (Ka/Ks value < 1, *p*-value Fisher’s exact test < 0.05) while only a single gene of unknown function (K06G5.1) was found to be under positive selection (Ka/Ks = 2.17, *p* = 0.03).

### Possible loss-of-function mutations

In order to understand the potential functional impact of the GVs in the Hawaiian strain, we divided impacted genes into three (potentially overlapping) categories (Table 2): (i) it is a single-copy gene (*i.e.* the gene has no annotated paralog in the *C. elegans* genome), (ii) it has a reported severe phenotype including lethality, sterility, sickness, developmental arrest, or other severe defects as detected by RNAi or genetics studies (hereinafter referred to as ‘severe phenotypes’), and (iii) it is orthologous to a human gene with OMIM annotation. Protein-coding genes in the first two categories can be regarded as essential. Genes that in addition map to human genes with associated diseases (via OMIM annotation) are highly conserved genes and good candidates for further genetics studies on deleterious alleles carried by individuals using *C. elegans* as a model organism.

**Table 2:** Categories of genes (rows) and their impact based on the detected GVs (columns)

	**Fully deleted**	**ORF disrupted**	**Radical + Mod radical SNVs**	**ORF impacted**
Total	93 (95)	1,128 (1,244)	2,586 (2,889)	7,828 (9,049)
Single copy	9 (9)	198 (234)	442 (525)	1,733 (2,081)
Single copy + lethal/sterile	1 (1)	36 (51)	113 (141)	429 (570)
Single copy + OMIM	0 (0)	3 (5)	12 (20)	57 (81)
Single copy + lethal/sterile + OMIM	0 (0)	1 (1)	7 (8)	28 (36)

Numbers in parenthesis correspond to the total spliced forms affected. ‘ORF Disrupted’ includes frame shift GVs, stop loss and gains, and GVs impacting splice sites. ‘ORF Impacted’ considers all types of GVs except synonymous SNVs.

Overall, we found that 22% (1,733 genes) of the 7,828 *C. elegans* genes impacted by some kind of GV other than synonymous SNVs are single-copy genes, with 429 genes (~3% of the total set of *C. elegans* genes) presenting severe phenotypes (Table 2). Furthermore, 57 genes (0.3% of the total genes) map to human orthologs that are associated with diseases. This set of genes, which are interesting candidate mutations that should be verified by cDNA sequencing and genetic methods, provides a rich basis for understanding how healthy individuals of a same species can carry deleterious mutations on genes that can be considered essential for the organism.

Table 2 also shows that the number of essential and OMIM genes decreases as the predicted impact of GVs increases (from ORF Impacted to Fully Deleted). For example, of the 93 genes fully deleted, there are no genes falling into all three categories.

In contrast to the 93 genes found fully deleted in the Hawaiian strain compared to the N2 reference, there are 1,128 genes with their ORF disrupted. Of these, 198 genes are single-copy, with 36 having associated severe RNAi or genetic phenotypes (as defined above) based on WS210. Interestingly, 3 single-copy genes (corresponding to 5 spliced forms) have as homologs human genes with OMIM annotations (Table 3).

**Table 3:** Single-copy genes with OMIM annotations and ORF disrupted in the Hawaiian strain

**Gene name**	**Sequence name**	**Position of disruption (%)**	**Human homolog**
*aex-3*	C02H7.3a	99.1	MADD
	C02H7.3b	92.6	
*hid-1*	K02E10.2a	90.5	DYM
	K02E10.2b	93.1	
T20H4.5	T20H4.5*	90.6	NDUFS8

Since transcripts can be impacted differently, each transcript is listed. The location of the disruption is measured with respect to the length of the peptide as described in WormBase for N2 strain. Sequence names with a ‘*’ indicate genes associated with severe phenotypes as defined in the text.

Protein-coding genes with ORF preserved could see their functionality significantly reduced if a deletion removes a functional domain. Based on WS210 domain annotation, we found 112 genes with a deletion that overlaps at least partially with an annotated domain. Gene Y49F6A.1 is an example of a deletion that has a full and a partial domain removed while having its ORF preserved. It encodes a 966 aa protein, translation initiator factor 2C (elF-2C). This protein has two domains: a PAZ domain (319–439 aa) and a PIWI domain (585–911 aa). All residues between 302 and 817 aa are deleted, removing the PAZ domain and most of the PIWI domain. A previous study on feeding of dsRNA for RNAi across different wild isolates of *C. elegans*[33] found that the Hawaiian strain had a defect in germline RNAi as a result of multiple mutations in a gene *ppw-1* that contains the same domains. *ppw-1* in the Hawaiian strain has a 1 bp deletion that introduces an early stop codon upstream of the PAZ and PIWI domains. Thus the function associated to Y49F6A.1 may be silenced in the Hawaiian strain due to the loss-of-function mutations.

A third category of impact on protein-coding genes in addition to genes fully deleted and genes disrupted (shown in Table 2) refers to the presence of radical or moderately radical amino acid substitutions, according to the categorization provided by Li and colleagues based on Grantham scores [66,67]. These types of substitutions may have a significant impact on protein structure and hence function. There are 442 single-copy genes with such GVs, 113 of which are associated with severe phenotypes. Of course, the categorization based on Grantham scores can only serve as guideline for assessing the impact of missense SNVs on protein-coding genes and cases that are not regarded as radical by such categorization can still have a significant impact on genes. For example, a previous study [32] has shown that the molecular basis for the Hawaiian strain not following the so called temperature-size rule (where ectotherms mature at a larger size at lower temperatures) corresponds to a transition from A to G on a DII-A domain of gene *tra-3*, generating a mutation from phenylalanine to leucine; this amino acid substitution is regarded as conservative by Li’s categorization.

Based on the set of single-copy genes with radical SNVs presenting severe phenotypes, we have selected and validated experimentally four radical SNVs by PCR amplification followed by DNA sequencing (Additional file 18).

### Impact of GVs on multi-gene families

The impact of GVs on protein-coding genes can be significant not only by impacting single-copy genes, but also by impacting multiple members of a same gene family. We explored how the four different levels of impact on protein-coding genes defined above affect different gene families (Table 4). Since there are many gene families, only those 30 most disrupted with at least 20 members are shown here, whereas the complete list can be found as (Additional file 19). In general, the gene families most impacted by GVs are those involved in protein-protein interactions and sensory mechanisms such as the MATH/BTB (represented by bath, math and btb names in Table 4), FBOX (represented by fbxa, fbxb and fbxc) and chemoreceptor genes.

**Table 4:** Top 30 gene families and the overall impact of GVs

	**Total members**	**Fully deleted**		**ORF disrupted**		**Radical + Moderately radical SNVs**		**ORF impacted**	
		**Number**	**%**	**Number**	**%**	**Number**	**%**	**Number**	**%**
btb	21	0	0.0	5	23.8	10	47.6	21	100.0
math	49	2	4.1	14	28.6	28	57.1	36	73.5
bath	37	4	10.8	5	13.5	18	48.6	27	73.0
fbxb	110	3	2.7	23	20.9	43	39.1	83	75.5
fbxa	194	8	4.1	31	16.0	73	37.6	144	74.2
srbc	73	1	1.4	9	12.3	25	34.2	58	79.5
srw	114	3	2.6	14	12.3	41	36.0	86	75.4
srz	66	2	3.0	9	13.6	20	30.3	48	72.7
oac	58	0	0.0	8	13.8	20	34.5	41	70.7
sri	60	0	0.0	3	5.0	21	35.0	44	73.3
fbxc	54	0	0.0	7	13.0	20	37.0	34	63.0
srh	222	6	2.7	15	6.8	63	28.4	139	62.6
srj	39	0	0.0	3	7.7	9	23.1	27	69.2
clec	256	2	0.8	31	12.1	70	27.3	143	55.9
cyp	76	0	0.0	5	6.6	20	26.3	48	63.2
sdz	36	0	0.0	6	16.7	10	27.8	17	47.2
srx	106	0	0.0	11	10.4	25	23.6	56	52.8
sre	52	0	0.0	3	5.8	9	17.3	32	61.5
scl	25	0	0.0	4	16.0	4	16.0	13	52.0
srab	23	0	0.0	2	8.7	6	26.1	11	47.8
set	32	0	0.0	1	3.1	6	18.8	18	56.3
nhr	278	0	0.0	18	6.5	52	18.7	146	52.5
srv	31	0	0.0	3	9.7	7	22.6	14	45.2
srt	66	0	0.0	7	10.6	11	16.7	33	50.0
npp	22	0	0.0	1	4.5	5	22.7	11	50.0
str	193	1	0.5	12	6.2	41	21.2	90	46.6
srg	62	0	0.0	3	4.8	9	14.5	30	48.4
gcy	32	0	0.0	1	3.1	3	9.4	17	53.1
pqn	72	0	0.0	2	2.8	11	15.3	33	45.8
tag	137	0	0.0	8	5.8	17	12.4	61	44.5

Only gene families larger than 20 genes were considered for this table. Gene families sorted by average percentage of gene family members impacted by GVs in each category. Full table is available as (Additional file 19: Table S5).

## Discussion

In this study we have chosen to compare two wild isolates of *C. elegans*: the N2 strain, isolated from Bristol, England in the 1950s by L.N. Staniland [4] and the CB4856 strain, also known as the Hawaiian strain, extracted from a pineapple field in Hawaii in 1972 [17]. These two strains present a number of differences in biological and behavioural traits including copulatory plug formation [17,28], intake of O_2_ and CO_2_[29-31], temperature-size rule [32], germline RNAi [33], response to benzaldehyde [34], thermal migration [35], pathogen susceptibility [36], biofilm resistance in the presence of *Yersinia*[37], and social behaviour and food response [38,39]. As well, other studies have shown no differences for other traits, such as sensitivity to supplemental zinc [71].

### Combined strength of long (Roche 454) and short (Illumina GA) reads

We have sequenced the CB4856 strain using Roche/454 and Illumina GA platforms. Alignment of the reads against the N2 reference strain and subsequent detection of GVs reveals hundreds of thousands of SNVs and small InDels, and thousands of large deletions and insertions.

Detection of SNVs and small InDels by these two different platforms demonstrated its complementary power; whereas Illumina GA provides a significant depth (67× in this case) useful for resolving many SNVs and small InDels, the length of 454 reads allows for the detection of these GVs in highly polymorphic regions. Such regions were known to exist between these two strains from previous studies [50], justifying our decision of sequencing this genome with those two platforms.

Even though there is an overall good agreement of SNVs and small InDels found in this study and those available in WormBase [20,21], the high presence of small InDels in homopolymeric regions generates a large disagreement between Illumina GA and the Roche/454 sequencing technologies. Homopolymers are a known issue for the Roche/454 platform specially for runs of 7 bps or larger [52]. A previous study on the genomic distribution of homopolymers in *C. elegans* reported close to 150,000 such regions of 8 bps or larger, with a chromosomal distribution that resembles that found for small InDels in this study, *i.e.*, a higher accumulation in the arms of autosomes [72]. We have observed SNVs and indels occur more frequently in the arms of autosomes than the center and more uniformly distributed in the X chromosome. Gene density is a likely factor that contributes greatly to the observed pattern where a greater gene density is found in the central cluster while it is more gene sparse in the arms [73]. The greater gene density in the centre of the chromosome would also have more essential genes [74]. The presence of higher essential gene content provides a selective pressure against mutations. On the other hand, X chromosome is known to contain very few essential genes [75] which could explain the more uniform SNV and InDels pattern.

In addition to SNVs and small InDels, we have found 1,430 large simple deletions in the Hawaiian genome compared to the N2 reference genome, 706 large deletions associated with type-A insertions, 254 type-A insertions associated with deletions, 57 type-B insertions and 473 type-B insertions associated with deletions.

### Advantage of DNA sequencing-based methods for detecting GVs over CGH

**Figure 10:**
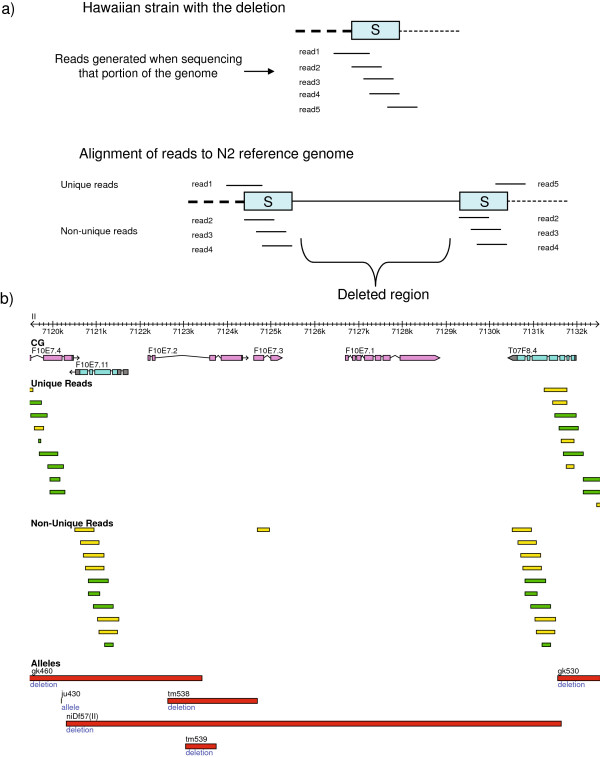
**NAHR-based deletions. a)** Illustration of NAHR-based deletions. A region of the Hawaiian genome is sequenced generating reads 1 to 5. These reads span a segment that we call ‘S’, that corresponds to the anchoring segment that generated a previous NAHR event. Once reads 1 to 5 are sequenced in the Hawaiian strain, the alignment of these to the N2 reference strain are such that those within the ‘S’ segment map non-uniquely (reads 2, 3 and 4), whereas those encompassing regions outside of ‘S’ should map uniquely to the genome (reads 1 and 5). The deleted region in the Hawaiian genome is expected to have no coverage in the N2 reference genome. **b)** A deletion generated by a NAHR event. This deletion is reported as niDf57 (II) by WormBase, based on the study of [48]. Reads in green indicate those aligned on the positive strand, whereas reads in yellow indicate those aligned on the negative strand.

A previous survey of deletions in the CB4856 genome using oligonucleotide array Comparative Genomic Hybridization (oaCGH) predicted 131 deleted regions in the Hawaiian genome compared to the N2 genome [48]. These deletions (hereafter called niDf deletions, as named in WS210) represent a 2% of the *C. elegans* gene set and range from 219 bps to 174.7 kbp in length (Additional file 20). Close inspection of these deletions shows that the majority (79) of the 131 niDf deletions are confirmed in this study; for all of these 79 niDf deletions we are able to define breakpoints at the base pair resolution (Additional file 20). Interestingly, for 11 of these 79 confirmed deletions we did not find a pattern of breakpoints as expected from the large simple deletions or the deletions associated with insertions. Instead, a pattern of non-unique reads aligning at the boundaries of these deletions suggests that they are generated by a non-allelic homologous recombination (NAHR) event (Figure 10).

In a previous study, we reported a 108 kb segmental duplication to be polymorphic among different laboratory strains [76]. During that study we also tested the Hawaiian strain for the presence of such duplication, revealing that it was absent. Inspection of the aligned reads to the genomic region harbouring the duplicons confirms the model stated in our previous work, where the duplication event was generated by NAHR (Additional file 21).

**Figure 11:**
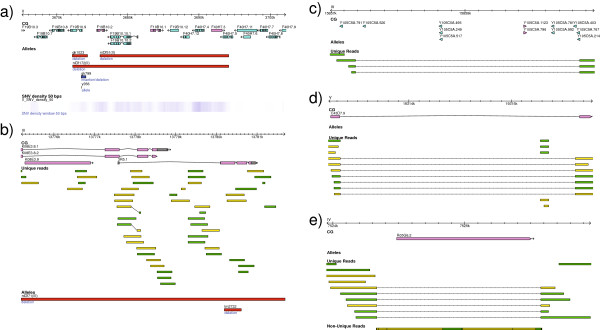
**Previously reported large deletions found to be false positives, and missed large deletions. a)** High density of SNVs within a previously reported deletion (niDf51). **b)** A previously reported deletion (niDf71) covered by unique reads. **c)** A deletion of a cluster of ncRNA genes. **d)** Deletion of an intron of a gene. **e)** Deletion of a *Cemar1* transposon, covered by non-unique reads due to its duplicative nature. **c)**, **d)** and **e)** are missed by a protein-coding exon-based oaCGH approach. Reads in green indicate those aligned on the positive strand, whereas reads in yellow indicate those aligned on the negative strand.

We have found that 29 of all 131 niDf deletions are likely false positives. Almost all (26 of 29) of these false positive deletions are caused by the very high incidences of SNVs within the genomic regions, which inhibit successful hybridization of probes designed based on the reference N2 genome sequences (Figure 11a and 11b).

It is worth mentioning that close inspection of the niDf deletions showed that many of them are likely *partially* false positives due to the high presence of SNVs and small InDels that co-occur with true deletions, generating an overestimation of the deleted regions in the Hawaiian strain. This, together with the likely false positives described above, explains why, whereas there is a very good agreement on the gene families most impacted in the Hawaiian strain by our study and that of Maydan and colleagues [48], the number of deleted members per family in their study is much higher than in our case.

Also, compared to the oaCGH, which relies on DNA hybridization, our approach using sequence alignment not only defines the exact deletion boundaries at base pair resolution, but also detects deletions in regions such as those containing ncRNA genes (Figure 11c), intronic/intergenic regions (Figure 11d) and those of duplicative nature (Figure 11e) such as transposable elements, that we have shown represent a significant part of the large deletions found in this study. Also, small deletions (smaller than 100 bps) are not reported by the oaCGH study, which, as we have seen, are the most prevalent. Hence, the number and impact of deletions across different regions of the genome is much more significant than previously reported.

### variationBlast and larger GVs

In addition to large deletions, in this study we have provided a successful methodology for the detection of large insertions based on convergent reads at the same breakpoint. These insertions, which we call type-B insertions to distinguish them from the type-A insertions that can be found within the length of the reads, are of unknown content and length without further computational detection based on assemblies or directly through experimental molecular techniques such as PCR. Attempts to detect larger insertions are necessary for a more accurate estimation of the GVs; for example, an accurate analysis of the activity of transposable elements (which are larger than 1 kb) between these two strains would only occur if all the instances of insertions of any length in the Hawaiian genome were to be found.

We have validated experimentally three type-B insertions and eight type-B insertions associated with deletions, with one extreme case of a 10 kb type-B insertion associated with deletion found in the Hawaiian genome compared to the reference N2 genome. Since the length and content of type-B insertions are not known, we did not include them as part of the overall impact of GVs on the protein-coding genes.

Interestingly, of the seven type-B insertions impacting seven protein-coding genes, two are unique genes (F10D2.8 and Y51A2D.7b) based on WS210. Furthermore, gene Y51A2D.7b displays phenotypes of sterility and embryonic lethality in RNAi trials [77-80]. In the same way, the deletions of the 473 type-B insertions associated with deletions impact 37 genes, 7 of which are single-copy and one of which *sdz-24* has an associated severe phenotype [77,79]. These results suggest that the GVs involving Type-B insertions could still have detrimental consequence in the Hawaiian worm.

### Confirming known GVs associated with trait differences

There are multiple previous studies that have shown behavioural and biological traits that are different between the N2 and the CB4856 strains. Many of these differences have an identified genetic basis. For example, a missense mutation of G > T at coordinate X:4,768,758 in gene *npr-1* generates an F215V codon change (from N2 to CB4856, respectively). This mutation has been associated with differences in the response to CO_2_ and O_2_[29-31], social behaviour and food response [38,39] and susceptibility to pathogens [36]. Results from a recent study on the origin of the 215 V allele, suggest that this allele arose during laboratory domestication of the N2 strain [29] and hence would not be an actual difference between the wild N2 and Hawaiian isolates.

Table 5 provides a summary of reported GVs between CB4856 and the N2 strain, the genes implicated and the difference in trait, if applicable. As shown in the table, we confirm most of the GVs reported. Still, some GVs are not necessarily found by our pipeline but are confirmed after inspection of the affected regions. For example, the reported deletion of an exon for gene *glb-5* in the Hawaiian strain compared to the N2 strain presents a clear pattern of a NAHR-based deletion (Additional file 22). This deletion is associated with differential responses to CO_2_ and O_2_[29,30].

**Table 5:** Comparison of published genotypes and associated phenotypes with GVs detected in this study

**Genes impacted**	**Published data**			**Our genomic analysis**		
	**Genotype (GVs)**	**Phenotype (Trait)**	**Reference**	**Result in our dataset**	**Coordinates**	**Observations**
*npr-1*	SNV;G > T;V215F	Intake of CO2/O2; Social behavior and food response; Pathogen susceptibilty	[29,36,38]	Found	X:4768758	N.A.
*glb-5*	Deleted 5th exon	Intake of CO2/O2	[29,30]	Found after inspection	V:5562441..5562810	NAHR-based deletion
*plg-1* (no gene model associated)	Deletion of LTR-Retrotransposon (retr-1; F44E2.2)	Copulatory plug formation	[28]	Found after inspection	III:8852505..8861364	Repeat at boundary
C49G7.1, D1065.3	2.9 kbp deletion	N.R.	[48]	Found	V:4057628..4060567	N.A.
*gst-38*	Multiple SNVs	N.R.	[48,50]	Found	V:15915782, 15915630, 15915570, 15915519, 15915351, 15915441, 15915316, 15915620, 15915480, 15915498, 15915561, 15915489, 15915347, 15915780, 15915777, 15915687, 15915318, 15915672, 15915666, 15915284, 15915393, 15915624, 15915837, 15915879, 15915417, 15915387, 15915439	N.A.
*tra-3*	SNV;T > C;F96L	Temperature-size rule	[32]	Found	IV:14442336	N.A.
*ppw-1*	SNV;T > C;F35S	Germline RNAi	[33]	Found	I:4186589	N.A.
	Insertion;ATT			Found after inspection	I:4186704..4186705	N.A.
	SNV;A > G;T245A			Found	I:4187463	N.A.
	Deletion;C			Found	I:4187632	N.A.
	SNV;C > T;L474L			Found	I:4188304	N.A.
	SNV;A > G;D691G			Found	I:4189045	N.A.
	SNV;A > G;K777E			Found after inspection	I:4189302	Falls in non-unique region
*tyra-3*	184 bps deletion	Patch-leaving	[81]	Found	X:4948657..4948841	Non-coding region
*zeel-1*	High divergence, 19 kb deletion	Required for compatibility between N2 and CB4856	[26]	Not found	N.A.	Divergent, complex region

In the case of gene *ppw-1*, associated to differences in germline RNAi [33], there are 7 GVs reported: 5 SNVs, one 3-bps insertion and one 1-bp deletion. The deletion generates a truncation of the protein short after its occurrence. Still, one of the reported variations downstream of that truncation, a SNV that generates a K777E codon change, is not found by our pipeline. Close inspection of the aligned reads shows that this SNV occurs in a non-unique region (Additional file 23), and since our pipeline focused only on the detection of GVs on reads uniquely aligned, then this GV was missed. Finally, another type of non-unique region, a repeat at the boundaries of the deletion spanning an LTR-retrotransposon within *plg-1*, associated with differences in copulatory plug formation [26], doesn’t allow for the immediate detection of the deleted LTR-retrotransposon (F44E2.2, or *retr-1*) with our pipeline.

These three examples (*glb-5*, *ppw-1* and *retr-1*) clearly illustrate that non-unique regions, even though challenging given the uncertainty of their duplicative nature, may contain important information regarding the impact of GVs on protein-coding genes can be missed.

A case that we could not confirm at the breakpoint resolution is the deletion associated with *zeel-1*[26], also listed in Table 5. This gene is found in a highly divergent 62 kb region spanning 2,317,000 and 2,379,000 in chromosome I (Additional file 24). Still, there is a ~19 kb region spanning gene *zeel-1* (Y39G10AR.5), in agreement with the work of Seidel and colleagues. The excessive number of SNVs and other small InDels at the breakpoints of the deletion suggests that some gaps might actually be regions so divergent that local alignment of a segment of a read is not possible, and hence two HSPs of a same read cannot be put together to report the large deletion.

Notably, a recent study by the Bargmann and Kruglyak groups [81] have shown that a deletion on a non-coding region of gene *tyra-3* is associated with differences in decision-making in *C. elegans* (Table 5); this result, which could not be found with strategies like the oaCGH, demonstrates the importance of detecting GVs genome-wide, and not only on protein-coding genes.

### Possible loss-of-function mutations

In addition to those studies that have reported a genetic basis for differences in traits, there are many reported differences between these two strains for which the genetic basis remains undefined. These include differences in response to benzaldehyde [34], thermal migration [35] and biofilm resistance in the presence of *Yersinia*[37]. Inspection of our detected GVs and their impact on protein-coding genes can shed light on the molecular basis that generates such differences. One example has to do with the response to benzaldehyde. A previous study shows that, after exposure to benzaldehyde in the absence of food, N2 displays a decreased attraction to that odorant whereas CB4856 fails to display decreased response [34]. We find that one single-copy gene, *gpc-1* (K02A4.2), which presents a benzaldehyde chemotaxis defective phenotype based on an RNAi experiment [82], and carries a radical missense SNV at coordinate X:12,882,299 (T > C) that generates a C12R codon change. This radical SNV might have a significant impact on the structure and function of the protein associated to *gpc-1*, and hence it is a good candidate for further studies that explore the genetic basis of the differential response to benzaldehyde.

In addition to the contribution that this dataset of discovered GVs might have on differential traits with unknown genetic basis, we expect our dataset of detected GVs to be a contribution to those traits that might already have an explained genetic basis but for which further discoveries can be found, such as the deletion of the PAZ and PIWI domains in the elF-2C reported earlier in this study, which might also be contributing to the differences in germline RNAi in addition to the truncation of *ppw-1*. Overall, we expect the set of GVs found in this study to be useful for further pursuing the genetic basis of these and other behavioural and biological trait differences between N2 and CB4856.

Several single-copy genes that have their ORF fully deleted or disrupted in CB4856 compared to N2 have also an associated severe RNAi or genetic phenotype (Table 6). We have found 37 genes with such features that are either fully deleted (1 gene) or have an ORF disrupted (36 genes). Furthermore, we found 429 genes (~2% of the total of *C. elegans* genes) that present those features and that are impacted by some kind of GVs (other than synonymous SNVs). If a gene that can be regarded as essential for a leaving organism is truncated, then the natural question is how is it possible that a healthy individual carries a mutation that is likely deleterious. Such apparent inconsistencies have also been observed in human individuals, for which current efforts of the 1,000 Genomes Project have shown that there are at least 100 loss-of-function (LOF) variants in the genome of a healthy human individual [83]. One explanation for such cases can be duplication events involving the genes in the Hawaiian strain compared to N2. This could be addressed by exploring the depth of the aligned reads compared to an average; an analysis of differences in copy-number based on read coverage goes beyond the scope of our study. Another explanation might be that we are dealing in many cases with a genetically complex system for which mutations in two or more genes balance each other, resulting in the preservation of fitness of the individual.

**Table 6:** Genes deleted or disrupted in Hawaiian strain that are associated with essential functions

**Sequence name**	**Gene name**	**Reference**	**Impact of GVs**
C29H12.5		[84,85]	ORF_DISRUPTED
F33C8.1	*tag-53*	[86]	ORF_DISRUPTED
K07E8.3	*sdz-24*	[77]	ORF_DISRUPTED
T28F12.3	*sos-1*	[87]	ORF_DISRUPTED
Y41D4B.11		[78]	ORF_DISRUPTED

## Conclusions

Our work confirms previously identified GVs associated with differences in behavioural and biological traits between the N2 and CB4856 strains and provides a rich resource for future studies that aim to explain the genetic basis for other trait differences.

## Methods

### Genome library and sequencing

Genomic DNA library was prepared from the Hawaiian strain following a standard protocol (http://genetics.wustl.edu/tslab/protocols/genomic-stuff/worm-genomic-dna-prep/) originally set up by the Andy Fire Lab. The library has been sequenced using the (i) Titanium 454 sequencing technology at the Genome Quebec Innovation Centre in one run, which yielded 1,237,732 reads, with an average length of 340 bps (median length of 372 bps), and (ii) Using Illumina GA sequencing technology at the Genome Science Centre in Vancouver, which yielded 85,494,844 paired-end reads of length 101 base-pair each.

### Read mapping

All reads were aligned against the WS210 version of the *C. elegans* genome. 454 reads were aligned using cross_match with default parameters, except for the min_score parameter that was set to 24 in order to increase sensitivity. Also, the parameter -masklevel 101 was set in order to report all high-scoring segment pairs (HSPs) to the reference genome for a given read. In order to increase speed, alignment was executed in parallel using the westgrid resource. Illumina reads were aligned using SSAHA2 [53] with the following parameters: -solexa, -pair 100,500, -align 0 –output sam_soft –mthreshold 20 –multi 0.

### Detection of GVs based on 454 reads

All HSPs from 454 reads generated with cross_match were provided as input to our newly developed tool called variationBlast. This program is built on an algorithm that is similar to that used for developing our gene prediction program genBlastA [88]. Briefly, a local sequence alignment tool (in this case cross_match) is used to find all local alignments between a sequence q (the read sequence) and r (the reference genome). There is no particular requirement on the type of aligner used, as variationBlast will be able to handle all kinds of alignments. Then, alignment results are converted into a format that is accepted by variationBlast, which reports a ranked list of reference regions that show homology to the read and annotate the SVs for each region as follows. Starting from a large number of unorganized local alignments between the read sequence and the reference genome, variationBlast detects SVs between the read sequence and the reference genome sequence in two steps. First, the local alignments (or HSPs) are filtered and organized into groups so that each group roughly corresponds to the entire read sequence. The groups are also ranked according to their similarity to the read. Second, for each group in the ranked order, variationBlast assembles the global alignment between the entire group and the read sequence based on the local alignments and reports SVs accordingly. The detailed algorithm will be published separately (manuscript in preparation).

In summary, variationBlast examines all HSPs for their relationship, groups the HSPs and annotates various types of GVs encountered. Specifically, variationBlast reports, for each read generating one or more HSPs, SNVs, insertions, deletions, transpositions and inversions. More importantly, variationBlast precisely defines base-pair level breakpoint coordinates for each type of GV. Since variationBlast has been designed to identify GVs using long reads, it will be increasingly useful as next-generation sequencing technologies point towards the generation of longer reads.

### Categorization of reads in unique and non-unique

For a given read used as query, if only one group is generated by variationBlast, or if the best group reported by variationBlast has a score which is at a distance of more than 2% from the score of the next group, then the read is considered unique. Otherwise, it is considered non-unique.

### VariationBlast SNV detection

Based on all SNV coordinates detected by variationBlast for each individual read, we defined a final set of SNVs based on the following criteria: (i) the coordinate is supported by at least two unique reads, (ii) there are no conflicting base pairs provided by other unique reads at the same coordinate, and (iii) the average quality is 30 or higher. For the matter of this study, SNVs are defined as substitutions only, not single base pair insertions or deletions.

### VariationBlast small insertions detection

Based on all insertion breakpoints detected by variationBlast for each individual read within a segment aligned locally with cross_match, we defined a final set of small insertions based on the following criteria: (i) the breakpoints of the insertion is supported by at least two unique reads, (ii) there are no conflicting unique reads aligning across any of the two breakpoints, (iii) the insertion doesn’t fall within a homopolymeric region (defined as the same base pair repeated 5 or more times), and (iv) for those insertions of length 1 bp, the average quality value of the nucleotides supporting the insertion is equal or higher than 30.

### VariationBlast small deletions detection

Based on all deletion breakpoints detected by variationBlast for each individual read within a segment aligned locally with cross_match, we defined a final set of small deletions based on the following criteria: (i) the breakpoints of the deletion are supported by at least two unique reads, (ii) there are no conflicting unique reads aligning across any of the two breakpoints, (iii) the deletion doesn’t fall within a homopolymeric region (defined as the same base pair repeated 5 or more times), and (iv) for those deletions of length 1 bps, the average quality value of the adjacent base pairs supporting the deletion is equal or higher than 30.

### Validation of SNVs and small InDels

Given the parameters set above for the detection of SNVs and small InDels, a randomly selected set of 40 such variants were selected for experimental validation, corresponding to 18 SNVs and 22 small InDels. Of the 18 SNVs experimentally assessed, all of them were validated (100% accuracy; Additional file 25). Of the 22 small InDels validated (12 insertions 10 deletions) only two of them found to be false positives, supporting a 95% overall accuracy in the predictions (Additional file 25).

Because of the stringent criteria we applied, a set of predicted SNVs and small InDels in the Hawaiian genome hosted in WS210 are not supported by our analysis. We examined the validity of this set of SNVs and small InDels by randomly testing 20 such variants (10 SNVs and 10 small InDels; Additional file 26). We found that, of 10 SNVs, all but one was experimentally validated, suggesting a 80% error rate; of the 10 small InDels (five insertions and five deletions), seven were experimentally validated while three were not validated, suggesting a 30% error rate. Taken together, the instances of SNVs and small InDels that are not supported by our analysis have high error rates. The validated cases of SNVs and small InDels were missed in our analysis due to stringent criteria. Further sequencing and analyses are thus needed to identify these variants.

### VariationBlast large deletion detection

Based on all large deletion breakpoints detected by variationBlast for each individual read, we defined a final set of deletions based on the following criteria: (i) the breakpoints of the deletion is supported by at least two unique reads, (ii) there are no conflicting unique reads aligning across any of the two breakpoints, (iii) within the candidate deleted region, there is no more than 50% of unique reads aligning to it, and (iv) the deletion doesn’t fall within a homopolymeric region (defined as the same base pair repeated 5 or more times).

### Definition of Type-A and Type-B insertions

Type-A insertions correspond to any unaligned segment of a read that is not a flanking region of the read. In contrast, Type-B insertions correspond to any flanking region of the read that is not aligned to the genome. This distinction is necessary since type-A insertions are limited by the length of the reads supporting it and hence of known length. Type-B insertions, on the other hand, can be much larger in size but of unknown length.

### VariationBlast large insertion detection

Based on all type-A and type-B insertion breakpoints detected by variationBlast for each individual read, we defined a final set of Type-A and Type-B insertions based on the following criteria: (i) the breakpoints of the insertion is supported by at least two unique reads, (ii) there are no conflicting unique reads aligning across any of the two breakpoints, and (iii) the insertion doesn’t fall within a homopolymeric region (defined as the same base pair repeated 5 or more times). Since Type-A and Type-B insertions from different reads can be supporting the same breakpoints, these were categorized as Type-A insertions [89].

### Detection of GVs based on Illumina reads

#### SNV and small InDel detection

SNVs and small InDels were detected using the pileup2snp and pileup2indel functions of VarScan v2.2.3 [54] with the following parameters: --min-coverage 20, --min-var-Freq 0.9, --min-avg-qual 30. Variants with more than 200× coverage were also filtered. Re-evaluation of the output was necessary for those coordinates that present 2 or more candidate SNVs. SAMtools [90] rmdup followed by pileup commands with default settings were used to generate the pileup necessary as input for VarScan.

#### Large deletion detection

Those Illumina reads that align partially to the reference based on the SSAHA2 alignment are potential cases of large deletions for which a large gap could not be introduced given SSAHA2s scoring scheme. These 24,057,890 reads were provided as input for running cross_match with default parameters, except for the min_score parameter that was set to 14 in order to increase sensitivity given the length of the read. Also, the parameter -masklevel 101 was set in order to report all high-scoring segment pairs (HSPs) to the reference genome for a given read. In order to increase speed, alignment was executed in parallel using the westgrid resource. All the HSPs were provided as input for variationBlast and reads were categorized as unique and non-unique, as done for the 454 reads. Based on all large deletion breakpoints detected by variationBlast for each individual read, we defined a final set of large deletions based on the following criteria: (i) the breakpoints of the deletion is supported by at least ten unique reads, (ii) the depth within the deleted region is less or equal than 10×, and (iii) the deletion is not found in the set of small InDels.

### Retrieval of WormBase WS210 GVs

#### WormBase WS210 SNVs

Based on the 123,492 SNVs for strain CB4856 retrieved from WormBase WS210 AceDB server, we filtered for those SNVs with (i) duplicated coordinates and (ii) with conflicting nucleotides involved with respect to the target (Hawaiian) or the reference (N2). This leaves a total of 116,999 SNVs.

#### WormBase WS210 small insertions

Based on the 1,557 insertions for strain CB4856 retrieved from WormBase WS210 AceDB server, we filtered those with (i) duplicated coordinates, and (ii) spurious (non-ACTG) sequences. This leaves a total of 1,543 insertions.

#### WormBase WS210 small deletions

Based on the 2,112 deletions for strain CB4856 retrieved from WormBase WS210 AceDB server, we filtered those with (i) duplicated coordinates, (ii) inconsistency between the reported length of the deletion and the actual sequence, and (iii) inconsistency between the reported deleted sequence and that found in WS210 for the same coordinate. This leaves a total of 2,086 deletions.

### Experimental validation of GVs

The candidate GVs were PCR amplified using the same genomic DNA library prepared from the CB4856 strain that was sent for whole-genome sequencing. For experimental validation, primers (Additional file 27) were designed in the flanking regions of the computationally identified GVs that are conserved between the N2 reference genome and the CB4856 genome (Additional file 27). The PCR amplification was performed using the home-made Taq polymerase, a kind gift from the Hutter Lab. For small InDels and SNVs the products were purified using the GE Healthcare Life Sciences GFX PCR DNA and Gel Purification Kit and submitted for sequencing (Macrogen, http://www.macrogen.com). For larger deletions and insertions, the validity was assessed based on the size of the bands on DNA electrophoresis gels.

## Abbreviations

GV: Genomic variation; CNV: Copy number variation; SNV: Single-nucleotide variation; SNP: Single-nucleotide polymorphism; InDel: Insertion and deletion; ORF: Open reading frame; RNAi: RNA interference; oaCGH: Oligonucleotide array comparative genomics hybridization; HSP: High scoring segment pair.

## Competing interests

The authors declare that they have no competing interests.

## Authors’ contributions

NC conceived this project. IAV and CF carried out all bioinformatics analysis. MTG, TZ, and ZQ experimentally validated genomic variations. JW prepared genomic DNA library. RS and KW developed variationBlast, with IAV, JSC and NC. IAV and NC wrote the manuscript, with input from all co-authors. All authors read and approved the final manuscript.

## Authors’ information

NC is a Professor at the Department of Molecular Biology and Biochemistry at Simon Fraser University, a Michael Smith Foundation for Health Research (MSFHR) Scholar, and a Canadian Institutes of Health Research (CIHR) New Investigator.

## Supplementary Material

Additional file 1 All SNVs. Click here for file

Additional file 2 Excluded SNVs. Click here for file

Additional file 3: Figure S1 Distribution of missense and non-sense SNVs along peptide sequences. Click here for file

Additional file 4 All small InDels. Click here for file

Additional file 5 Excluded small InDels. Click here for file

Additional file 6: Figure S2 Distribution of Illumina-InDels adjacent to homopolymers of varying length. Click here for file

Additional file 7: Figure S3 Length Distribution of small Illumina-InDels (top) and small 454-InDels (bottom). Click here for file

Additional file 8: Figure S4 Number of small InDels impacting exons vs other regions of the genome. **a)** Frequency among exonic and non-exonic regions. **b)** Length distribution of small exonic InDels (left) and small non-exonic InDels (right). Click here for file

Additional file 9: Figure S5 Distribution of disruptive small InDels along peptide sequences. Click here for file

Additional file 10 Large deletions. Click here for file

Additional file 11 Deleted transposons. Click here for file

Additional file 12 Type A insertions. Click here for file

Additional file 13 Type B insertions. Click here for file

Additional file 14 Deletions associated with type A insertions. Click here for file

Additional file 15 Type A_insertions_assoc_deletions. Click here for file

Additional file 16 Type B insertions associated with deletions. Click here for file

Additional file 17 Transcripts impacted by GVs, including information about whether transcripts are single-copy, known OMIM disease genes, associated RNAi and genetic phenotypes, numbers and types of mutations impacting transcripts, ka/ks values, and impacted domains. Click here for file

Additional file 18: Table S1 and Table S2 Validated small InDels and radical amino acid substitutions. Click here for file

Additional file 19: Table S5 Gene family members impacted by GVs. Click here for file

Additional file 20: Table S3 Assessment of previously reported large deletions based on aCGH. Click here for file

Additional file 21: Figure S6 Large polymorphic segmental duplication. The aligned Hawaiian reads support the model that the duplication event was due to NAHR of *Cemar1* transposable elements at the flanking regions. The ‘Triplicates non-overlap’ track displays the alignment of the same non-unique reads to the locations were the *Cemar1* transposons are located. Click here for file

Additional file 22: Figure S7 Deletion of an exon in *glb-5* is due to a NAHR event. Alignment of reads around the sixth exon of the ‘b’ spliced form displays a clear pattern of NAHR, as illustrated in Figure 10. For simplicity, the tracks for unique and non-unique reads are displayed in compact mode. Reads in green indicate those aligned on the positive strand, whereas reads in yellow indicate those aligned on the negative strand. Click here for file

Additional file 23: Figure S8 A SNV found within a non-unique region. This SNV has been reported before for gene *ppw-1*. Click here for file

Additional file 24: Figure S9 Highly divergent region encompassing *zeel-1. zeel-1* is highlighted in yellow. Click here for file

Additional file 25: Table S4 Randomly selected SNVs and small InDels validated by PCR. Click here for file

Additional file 26 Randomly selected SNVs and small InDels retrieved from WS210 validated. Click here for file

Additional file 27: Table S6 Primer pairs used GV validation. Click here for file
