# Supplementary figures and images for: Genome-wide variations in a natural isolate of the nematode Caenorhabditis elegans
Source: BMC Genomics. 2014 Apr 2;15:255. doi: 10.1186/1471-2164-15-255 (PMC4023591; doi:10.1186/1471-2164-15-255)

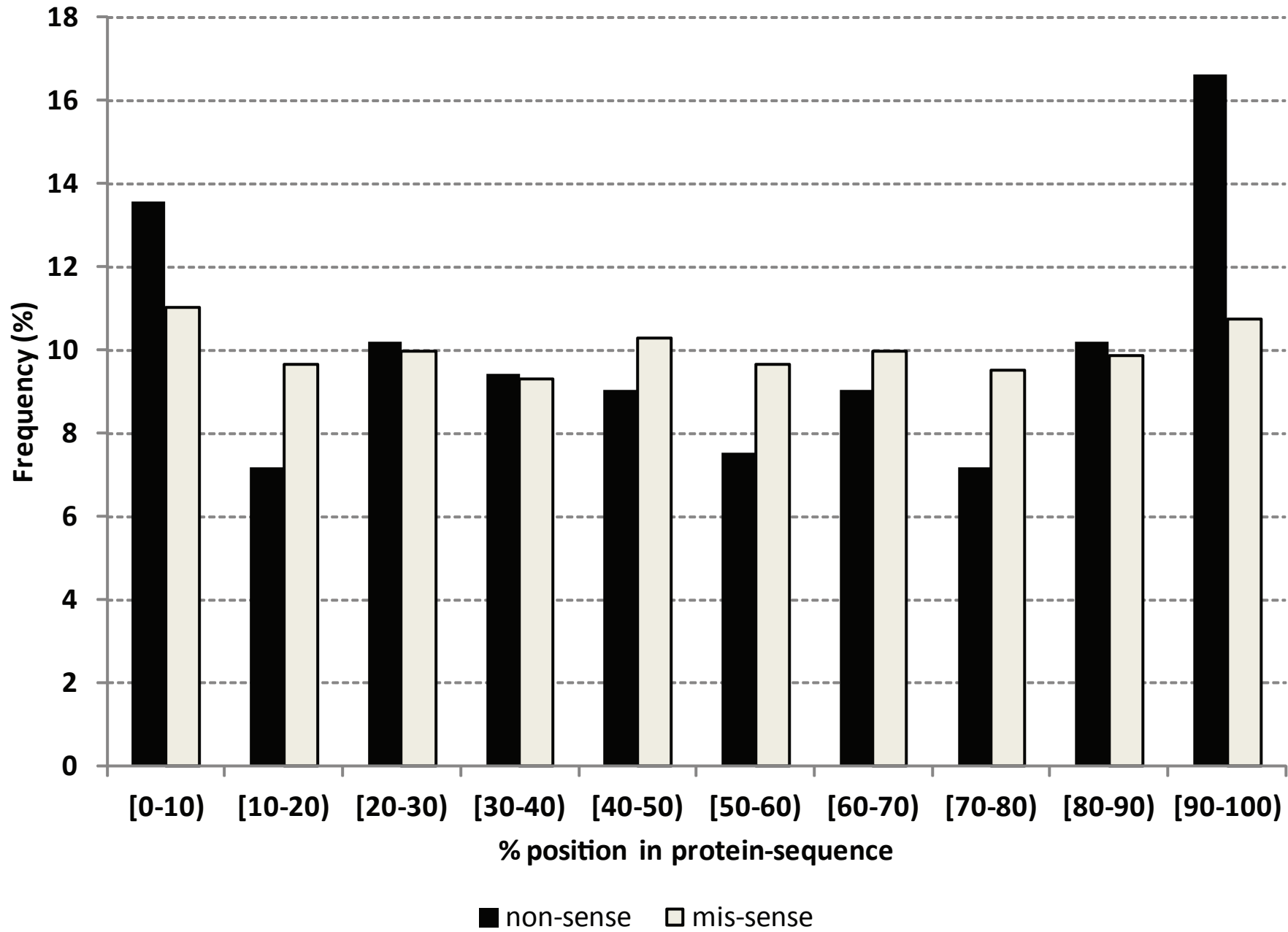

Supplement: Additional file 3: Figure S1 — Distribution of missense and non-sense SNVs along peptide sequences. [file 1471-2164-15-255-S3.pdf]

# **% Illumina small InDels in Homopolymeric Regions**

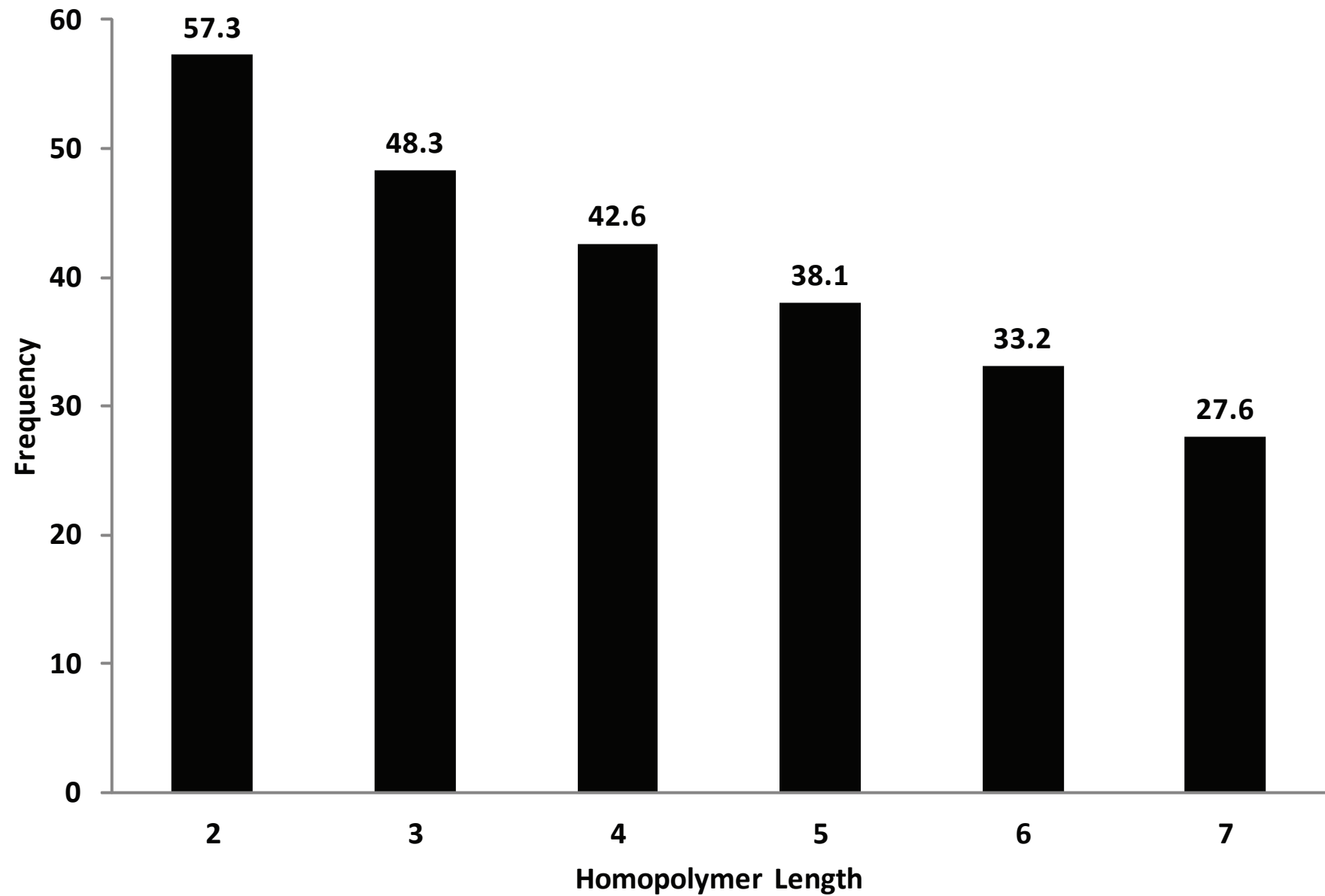

Supplement: Additional file 6: Figure S2 — Distribution of Illumina-InDels adjacent to homopolymers of varying length. [file 1471-2164-15-255-S6.pdf]

Length Distribution Illumina-InDels

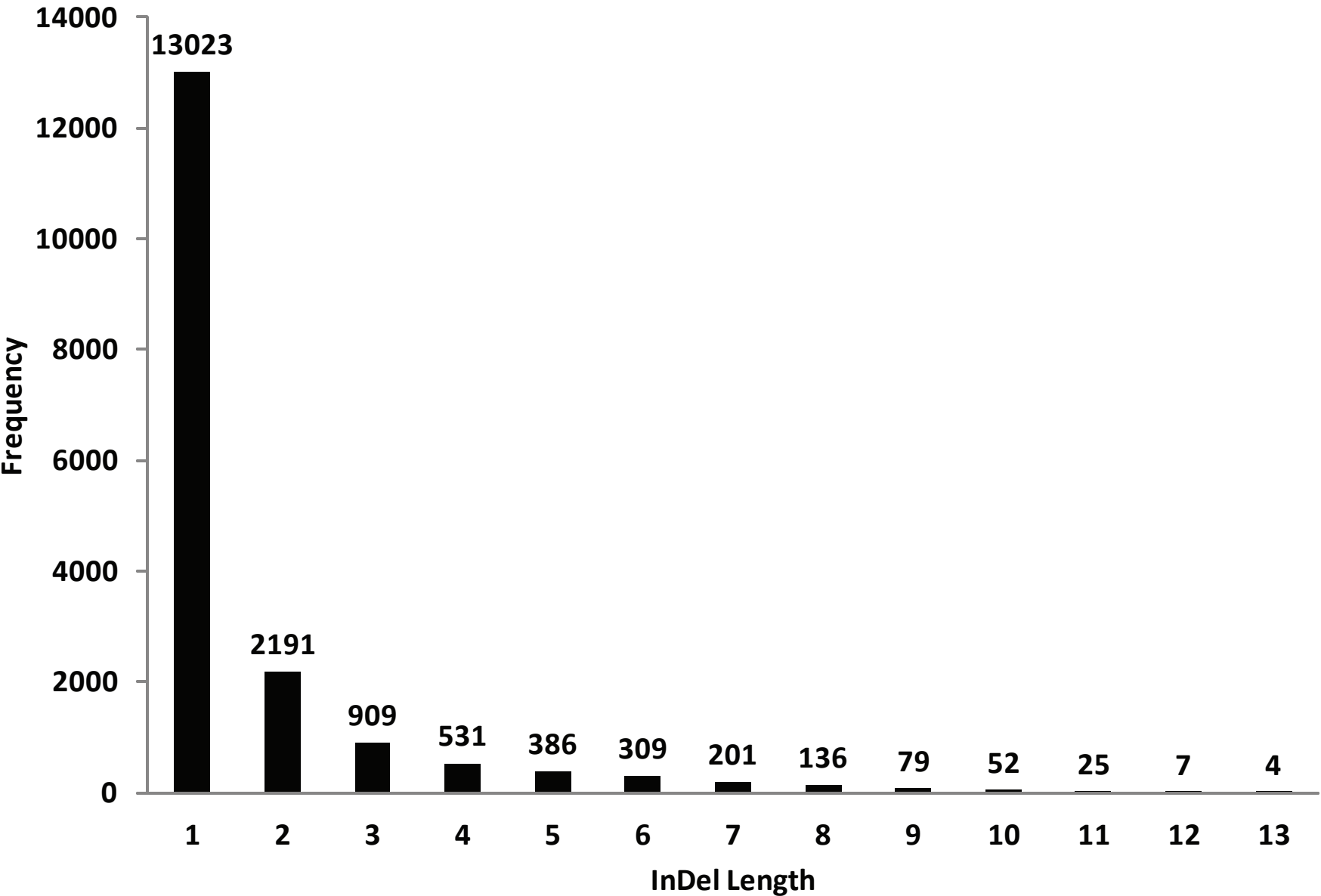

Length Distribution 454-InDels

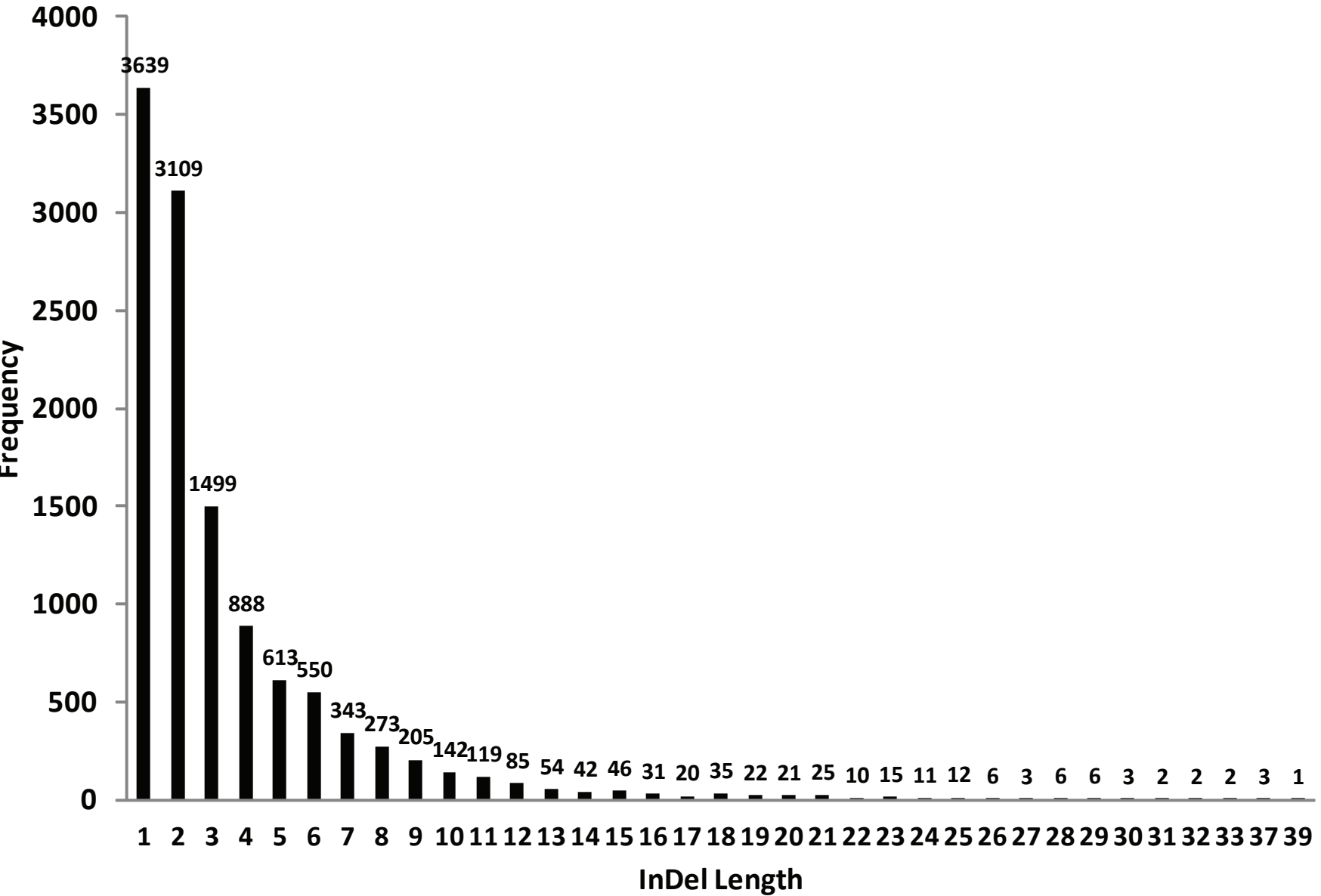

Supplement: Additional file 7: Figure S3 — Length Distribution of small Illumina-InDels (top) and small 454-InDels (bottom). [file 1471-2164-15-255-S7.pdf]

a)

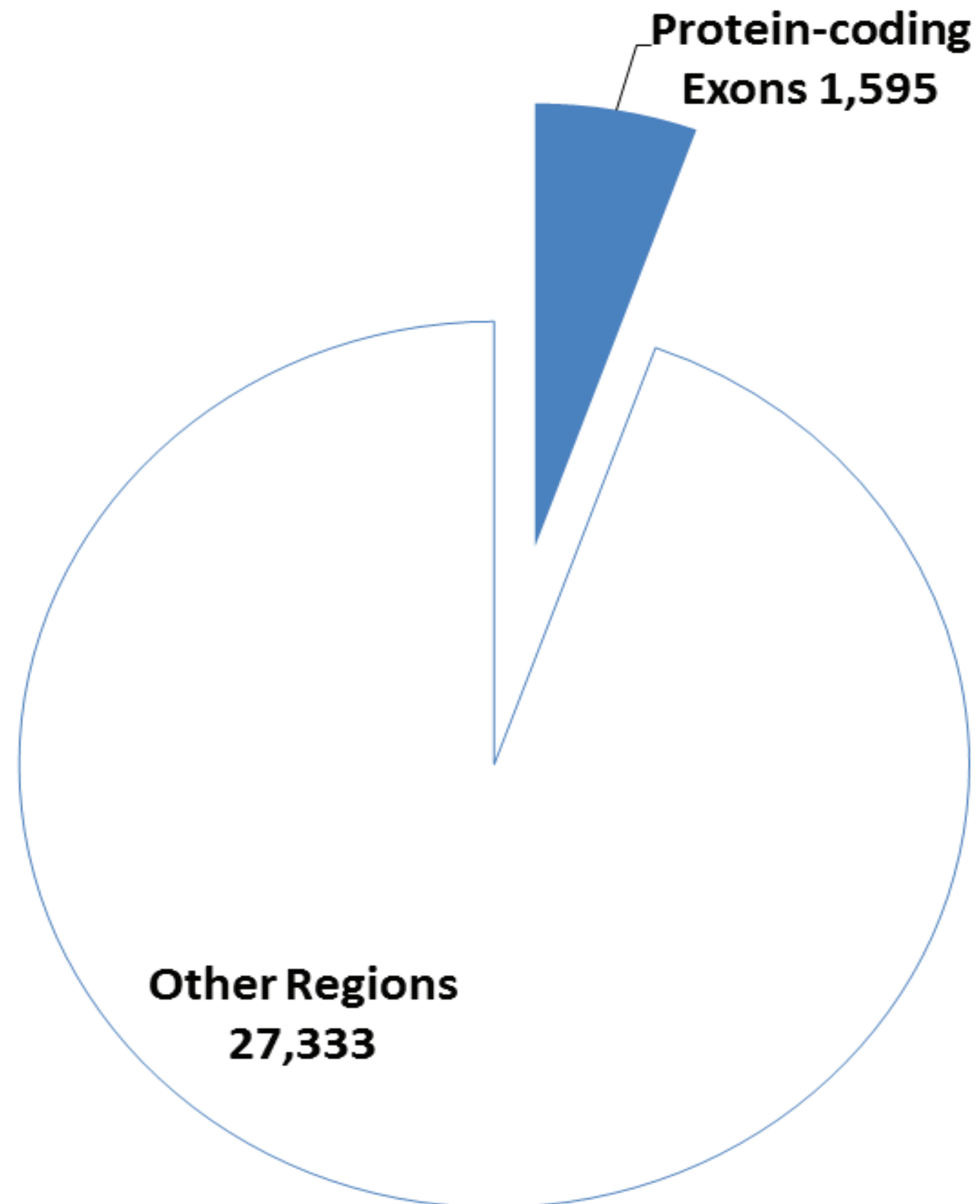

b)

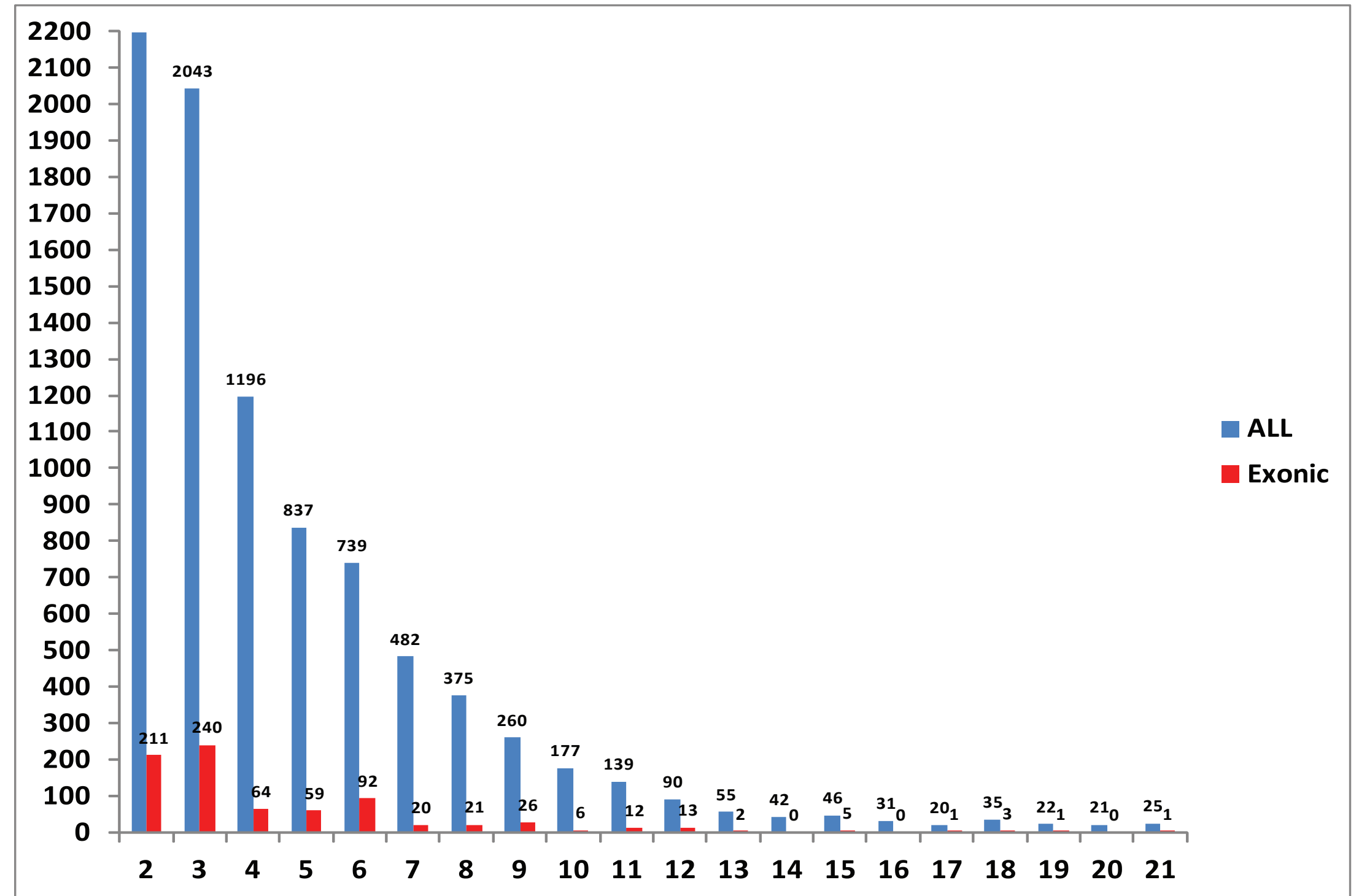

Supplement: Additional file 8: Figure S4 — Number of small InDels impacting exons vs other regions of the genome. a) Frequency among exonic and non-exonic regions. b) Length distribution of small exonic InDels (left) and small non-exonic InDels (right). [file 1471-2164-15-255-S8.pdf]

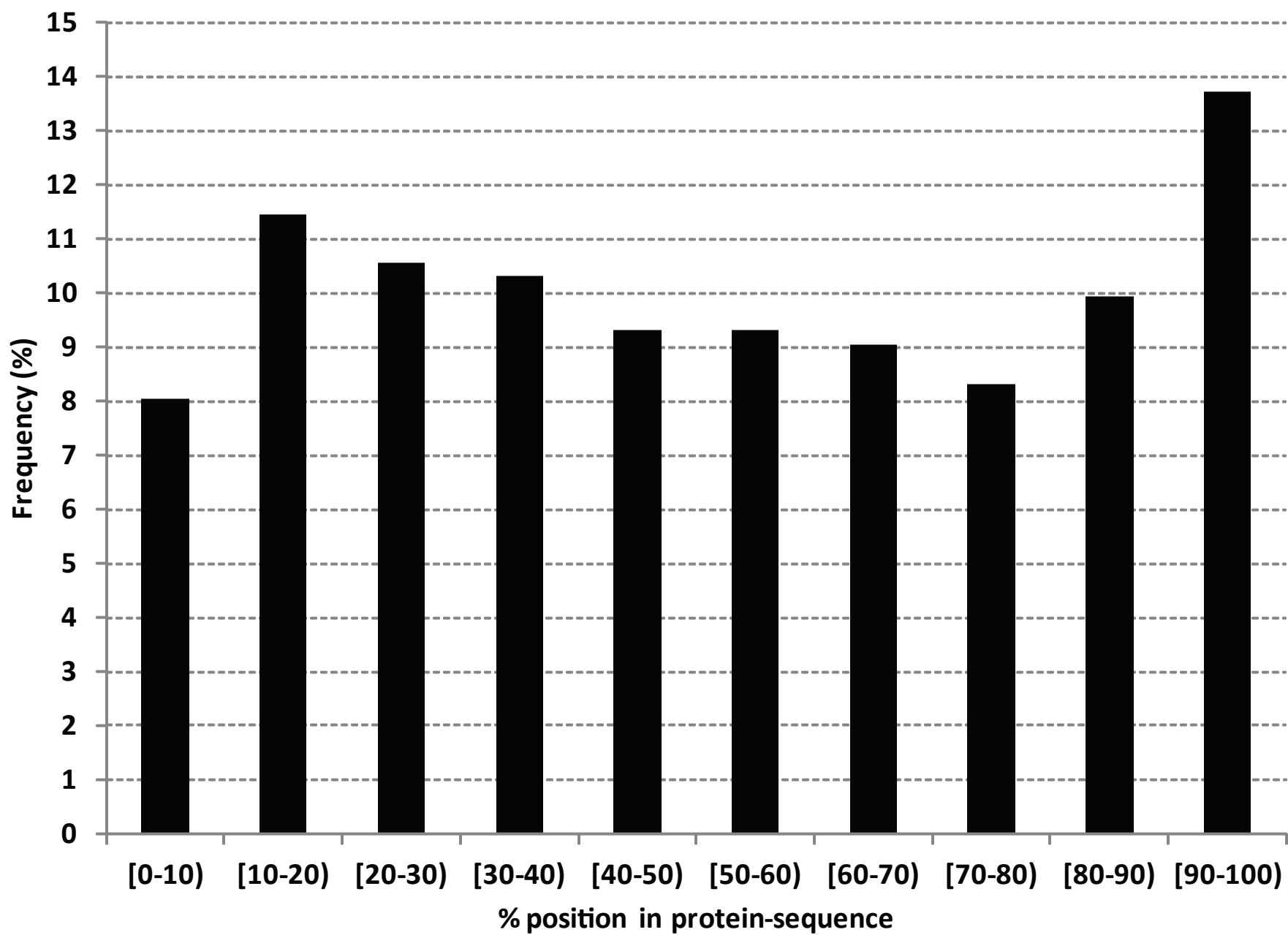

Supplement: Additional file 9: Figure S5 — Distribution of disruptive small InDels along peptide sequences. [file 1471-2164-15-255-S9.pdf]

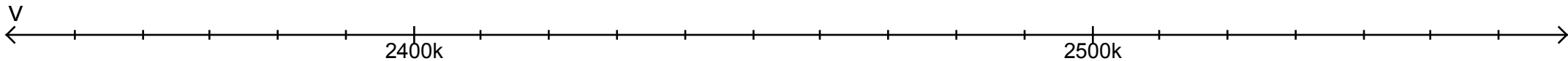

CG

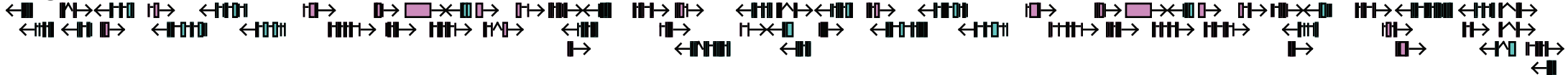

Unique Reads

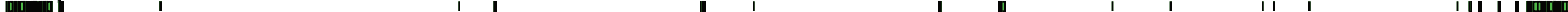

Non-unique Reads

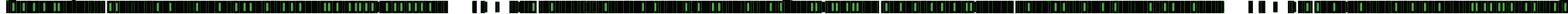

Triplicates non-overlap

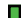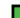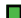

Supplement: Additional file 21: Figure S6 — Large polymorphic segmental duplication. The aligned Hawaiian reads support the model that the duplication event was due to NAHR of Cemar1 transposable elements at the flanking regions. The ‘Triplicates non-overlap’ track displays the alignment of the same non-unique reads to the locations were the Cemar1 transposons are located. [file 1471-2164-15-255-S21.pdf]

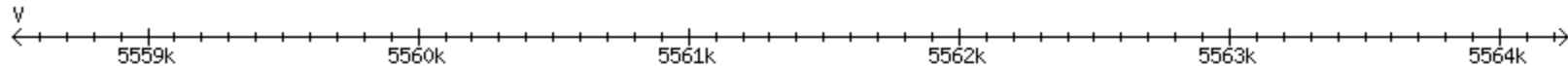

CG

C18C4.1b

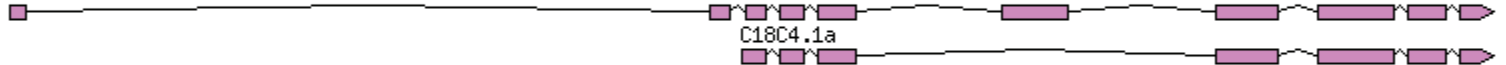

Unique Reads

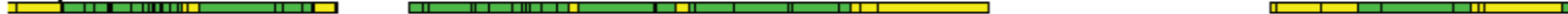

Non-unique Reads

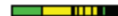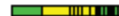

Supplement: Additional file 22: Figure S7 — Deletion of an exon in glb-5 is due to a NAHR event. Alignment of reads around the sixth exon of the ‘b’ spliced form displays a clear pattern of NAHR, as illustrated in Figure 10. For simplicity, the tracks for unique and non-unique reads are displayed in compact mode. Reads in green indicate those aligned on the positive strand, whereas reads in yellow indicate those aligned on the negative strand. [file 1471-2164-15-255-S22.pdf]

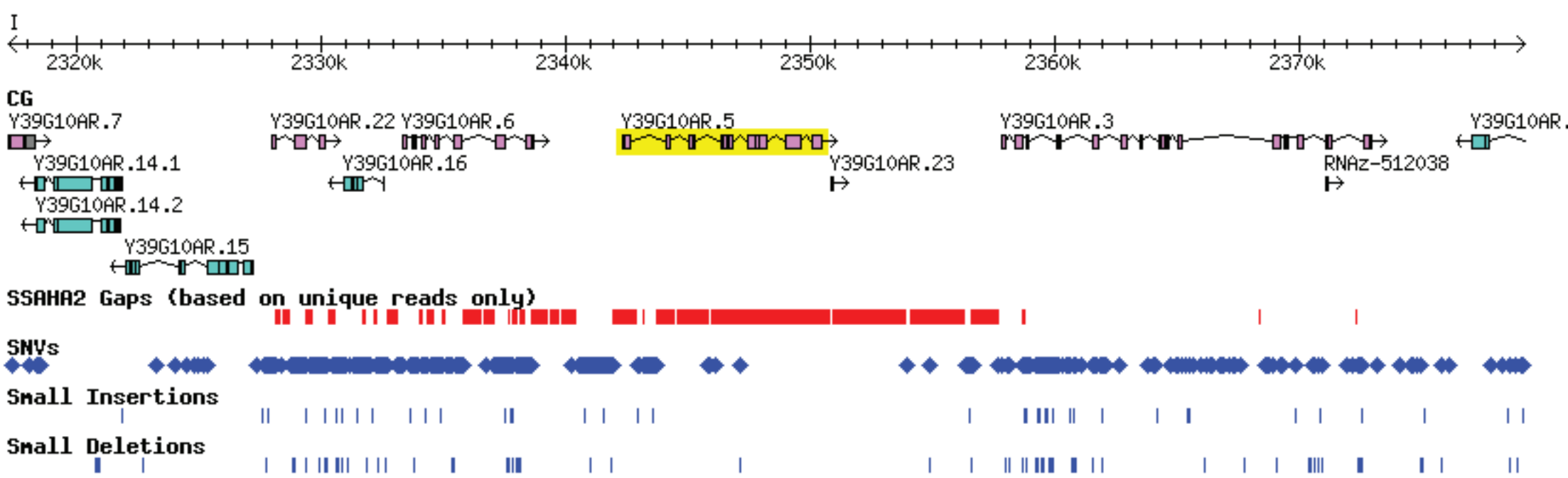

Supplement: Additional file 24: Figure S9 — Highly divergent region encompassing zeel-1. zeel-1 is highlighted in yellow. [file 1471-2164-15-255-S24.pdf]
