# Supplementary material for: Genome-wide variations in a natural isolate of the nematode Caenorhabditis elegans
Source: BMC Genomics. 2014 Apr 2;15:255. doi: 10.1186/1471-2164-15-255 (PMC4023591; doi:10.1186/1471-2164-15-255)
Supplement: Additional file 18: Table S1 and Table S2 — Validated small InDels and radical amino acid substitutions. [file 1471-2164-15-255-S18.doc]

Supplementary Table 1. Small InDels validated.

| Gene Name | GVs | Sequence | Coordinate | Single-Copy? | RNAi let/ster? | ORF Impact |
| --- | --- | --- | --- | --- | --- | --- |
| Y40B1A.1 | 1 bp del | G | I:13345365 | Y | Y | DISRUPTED |
| *tsr-1* (F53G2.6) | 1 bp ins | C | II:2471343..2471344 | Y | Y | DISRUPTED |
| Y45F10D.7 | 1 bp del | C | IV:13791001 | Y | Y | DISRUPTED |
| *sdz-28* (R52.1) | 1 bp ins | T | II:2131318..2131319 | N | Y | DISRUPTED |

Supplementary Table 2. Radical amino acid substitutions validated.

| Gene Name | Function/Domain | Coordinate | Subs AA |
| --- | --- | --- | --- |
| *tac-1* | TACC (transforming acid coiled coil) | II:14753433 | C94W |
| K01G5.10 | Hydrolase | III:10757600 | Y237D |
| *sec-3* | yeast SEC homolog | X:3096875 | F628S |
| R07R5.1 | RNA binding protein, G-patch domain | III:4410066 | V296D |
