# Supplementary material for: Genome-wide variations in a natural isolate of the nematode Caenorhabditis elegans
Source: BMC Genomics. 2014 Apr 2;15:255. doi: 10.1186/1471-2164-15-255 (PMC4023591; doi:10.1186/1471-2164-15-255)
Supplement: Additional file 23: Figure S8 — A SNV found within a non-unique region. This SNV has been reported before for gene ppw-1. [file 1471-2164-15-255-S23.pdf]

WS210 gene models

C18E3.7a

C18E3.7c

C18E3.7c

C18E3.7d

C18E3.7d

C18E3.7d

Hawaiian Unique Illumina Reads SSAHA2

3:110:3805:5220:W/3

a c a a a t

3:21:9307:12361:Y

a c g a a t

3:39:17013:15505:Y/3

a c g a a t

Hawaiian Non-unique Illumina Reads SSAHA2

a a a t
a c a a t
a c
a c a a a t
a c g a a t
a c
a c a a a t
a c g a a t
a c g a a t
a c a a a t
a c a a a t
a c a a a t
a c g a a t
a c g a a t
a c g a a t
a c a a a t
a c g a a t
a c a a a t
a c a a a t
a c g a a t
a c g a a t
a c g a a t
a c a a a t
a c a a a t
a c g a a t
a c g a a t
a c a a a t
a c g a a t
a c a a a t
a c g a a t
